# Underrecognized triploidy and genome-wide uniparental disomy in human blastocysts revealed by a concurrent preimplantation genetic testing approach

**DOI:** 10.1093/hropen/hoag044

**Published:** 2026-05-19

**Authors:** Ying Li, Matthew Hoi Kin Chau, Ying Xin Zhang, Bao Heng Gui, Wai Ting Lui, Ching Man Vivian Lam, Ye Cao, Teresa Cheuk Yan Chung, Chong Cong Wu, Patricia Nga Ping Ip, David Yiu Leung Chan, Yvonne Ka Yin Kwok, Jacqueline Pui Wah Chung, Kwong Wai Choy

**Affiliations:** Center for Reproductive Medicine, The Fifth Affiliated Hospital, Sun Yat-sen University, Zhuhai, China; Department of Obstetrics and Gynaecology, The Chinese University of Hong Kong, Hong Kong SAR, China; Department of Obstetrics and Gynaecology, The Chinese University of Hong Kong, Hong Kong SAR, China; The Chinese University of Hong Kong-Baylor College of Medicine Joint Center for Medical Genetics, Hong Kong SAR, China; Shenzhen Research Institute, The Chinese University of Hong Kong, Shenzhen, China; The Hong Kong Hub of Obstetric and Paediatric Excellence, The Chinese University of Hong Kong, Hong Kong SAR, China; Department of Obstetrics and Gynaecology, Fertility Preservation Research Center, The Chinese University of Hong Kong, Hong Kong SAR, China; Department of Obstetrics and Gynaecology, The Chinese University of Hong Kong, Hong Kong SAR, China; Department of Medical Genetics, Shandong Provincial Hospital Affiliated to Shandong First Medical University, Jinan, China; Prenatal Diagnosis Center, Shandong Provincial Hospital Affiliated to Shandong First Medical University, Jinan, China; Shenzhen Research Institute, The Chinese University of Hong Kong, Shenzhen, China; Center for Medical Genetics and Genomics, The Second Affiliated Hospital of Guangxi Medical University, Nanning, China; The Guangxi Health Commission Key Laboratory of Medical Genetics and Genomics, The Second Affiliated Hospital of Guangxi Medical University, Nanning, China; Department of Obstetrics and Gynaecology, The Chinese University of Hong Kong, Hong Kong SAR, China; Department of Obstetrics and Gynaecology, The Chinese University of Hong Kong, Hong Kong SAR, China; Department of Obstetrics and Gynaecology, The Chinese University of Hong Kong, Hong Kong SAR, China; The Chinese University of Hong Kong-Baylor College of Medicine Joint Center for Medical Genetics, Hong Kong SAR, China; Shenzhen Research Institute, The Chinese University of Hong Kong, Shenzhen, China; The Hong Kong Hub of Obstetric and Paediatric Excellence, The Chinese University of Hong Kong, Hong Kong SAR, China; Department of Obstetrics and Gynaecology, Fertility Preservation Research Center, The Chinese University of Hong Kong, Hong Kong SAR, China; Department of Obstetrics and Gynaecology, The Chinese University of Hong Kong, Hong Kong SAR, China; Center for Reproductive Medicine, The Fifth Affiliated Hospital, Sun Yat-sen University, Zhuhai, China; Department of Obstetrics and Gynaecology, The Chinese University of Hong Kong, Hong Kong SAR, China; Department of Obstetrics and Gynaecology, The Chinese University of Hong Kong, Hong Kong SAR, China; Department of Obstetrics and Gynaecology, The Chinese University of Hong Kong, Hong Kong SAR, China; Department of Obstetrics and Gynaecology, The Chinese University of Hong Kong, Hong Kong SAR, China; Department of Obstetrics and Gynaecology, Fertility Preservation Research Center, The Chinese University of Hong Kong, Hong Kong SAR, China; Department of Obstetrics and Gynaecology, The Chinese University of Hong Kong, Hong Kong SAR, China; The Chinese University of Hong Kong-Baylor College of Medicine Joint Center for Medical Genetics, Hong Kong SAR, China; Shenzhen Research Institute, The Chinese University of Hong Kong, Shenzhen, China; The Hong Kong Hub of Obstetric and Paediatric Excellence, The Chinese University of Hong Kong, Hong Kong SAR, China; Department of Obstetrics and Gynaecology, Fertility Preservation Research Center, The Chinese University of Hong Kong, Hong Kong SAR, China

**Keywords:** preimplantation genetic testing, genome-wide uniparental disomy, triploidy, molar pregnancy, early miscarriage, single-nucleotide polymorphism, haplotyping, segregation error, reproductive genetics, ART

## Abstract

**STUDY QUESTION:**

What is the incidence of triploidy and genome-wide uniparental disomy (gwUPD) in human embryos currently undetected by conventional preimplantation genetic testing (PGT)?

**SUMMARY ANSWER:**

Triploidy and gwUPD were detected in 0.8% (8/1049) and 1.4% (15/1049) of blastocysts, respectively.

**WHAT IS KNOWN ALREADY:**

Concurrent PGT enhances embryo selection by simultaneously detecting monogenic disorders (PGT-M) or structural rearrangements (PGT-SR) and aneuploidy (PGT-A). However, triploidy and gwUPD, two genome-wide abnormalities causative of severe pregnancy outcomes, are not routinely detected by conventional PGT platforms and thus remain under-recognized at the preimplantation stage.

**STUDY DESIGN, SIZE, DURATION:**

This study consisted of three phases: Phase I validated the detection capability of PGT-Plus on cell lines with previously ascertained genetic abnormalities; Phase II performed retrospective clinical validation on leftover trophectoderm biopsy whole-genome amplification (WGA) samples, including 65 for PGT-M, 67 for PGT-SR, and 130 for PGT-A received during January 2019 and July 2023; and Phase III was prospective implementation of PGT-Plus under a diagnostic setting on 529 blastocysts received from August 2023 to June 2024. Additionally, a cohort consisting of 258 embryo transfers, previously ascertained as euploid by conventional PGT-A methods but associated with unfavorable outcomes, was retested by PGT-Plus, which identified previously undetected cases of gwUPD or triploidy.

**PARTICIPANTS/MATERIALS, SETTING, METHODS:**

In Phase I, cell lines with ascertained genetic abnormalities were tested using at least one of the following methods, including G-banded Karyotyping, quantitative fluorescence PCR (QF-PCR), chromosomal microarray analysis (CMA), and mate-pair sequencing. The previous results were compared with those from PGT-Plus. In Phase II, results from conventional PGT platforms were compared in parallel with PGT-Plus to evaluate its accuracy. Cell lines or real trophoblast biopsy samples from blastocysts were amplified by the multiple displacement amplification (MDA) method. PGT-Plus data analysis was performed in various dimensions. For PGT-A analysis, except for canonical read-count analysis to assess the genome dosage, single nucleotide polymorphism (SNP) analysis was also performed to identify triploidy and gwUPD. Furthermore, for families requesting PGT-M and PGT-SR, linkage analysis was carried out for haplotype construction in addition to read-count analysis and SNP analysis. In addition to prospective cohort of Phase III, a unique cohort with adverse reproductive outcomes after transfer of a ‘euploid’ embryo, as determined by conventional PGT-A platforms, was retested to identify any triploidy and UPD among these copy-number neutral embryos. The unfavorable outcomes included no pregnancy, biochemical pregnancy, miscarriage, and termination of pregnancy due to fetal anomaly.

**MAIN RESULTS AND THE ROLE OF CHANCE:**

PGT-Plus detected all abnormalities ascertained by prior platforms in Phases I and II. In total, triploidy and gwUPD were detected in 0.8% (8/1049) and 1.4% (15/1049) of blastocysts, respectively. Notably, 3.5% (9/258) of the unfavorable transfers of embryos previously classified as ‘euploid’ would have been prevented if triploidy and gwUPD were detected. In addition, 45.7% (12/35) of embryos previously classified as balanced by PGT-SR were translocation carriers.

**LIMITATIONS, REASONS FOR CAUTION:**

PGT-Plus, similar to other relative phasing methods, requires an additional family member for haplotype determination, although an embryo with a known genotype via prior independent testing is also acceptable. In addition, short-read next-generation sequencing (NGS) impedes the capacity of PGT-Plus to directly detect pathogenic SNVs located in highly repetitive regions of the genome, e.g. the *HBA1* gene causative of thalassemia, which still require conventional methods, including short tandem repeats and PCR, to safeguard clinical diagnosis or can be addressed by long-read sequencing.

**WIDER IMPLICATIONS OF THE FINDINGS:**

This newly established PGT-Plus approach additionally detected gwUPD and triploidy in preimplantation embryos. It explained another 3.5% of ‘euploid’ embryo transfer failures. Meanwhile, embryo selection was enhanced by integrating PGT-M, PGT-SR, or PGT-A with an analysis of parental origin and stage of non-disjunction within a single PGT-Plus workflow.

**STUDY FUNDING/COMPETING INTEREST(S):**

This work was funded by the Research Grant Council Collaborative Research Fund (grant no. C4062-21GF to K.W.C.), the National Natural Science Foundation of China (grant no. 82502033 to Y.L.), and the Research Grant Council General Research Fund (grant no. 14100822 to J.P.W.C.). All authors declare that they have no conflicts of interest.

**TRIAL REGISTRATION NUMBER:**

N/A.

WHAT DOES THIS MEAN FOR PATIENTS? Preimplantation genetic testing (PGT) of embryos during IVF has been an essential component of assisted reproductive treatment (ART) as a means of avoiding the transmission of certain types of genetic abnormalities to future offspring. However, the current PGT systems are unable to detect triploidy (where the embryo has three instead of two copies of every chromosome) or genome-wide uniparental disomy (gwUPD; where two copies of every chromosome come from one parent and no copy comes from the other), despite both of these conditions having significant harmful consequences on embryo development and human reproduction. Identifying these anomalies at the preimplantation level could lower the risk of hydatidiform moles, miscarriages, and pregnancy-related maternal complications, thereby improving overall reproductive outcomes.

This three-phase study tested a new system for PGT, namely PGT-Plus, which detects triploidy and gwUPD; these abnormalities were found to be not uncommon at the preimplantation embryo stage, being detected in 0.8% and 1.4% of embryos, respectively. Their detection could reduce unfavorable reproductive outcomes by avoiding the transfer of these high-risk embryos. Furthermore, embryonic triploidy and gwUPD, which evaded detection from conventional PGT platforms, explained 3.5% (9/258) of implantation failures and miscarriages following the transfer of previously misclassified yet apparently normal ‘euploid’ embryos; earlier detection of these anomalies would have prevented these outcomes.

PGT-Plus can also differentiate normal balanced embryos from those that carry balanced translocations (where there is normal chromosomal content but an abnormal rearrangement of chromosomes). In this study, 45.7% (12/35) of these apparently normal embryos were actually identified as translocation carriers.

Here, we present a new comprehensive PGT platform that maintains a similar performance to that of conventional platforms while additionally detecting triploidy and gwUPD. By addressing these critical gaps, PGT-Plus empowers IVF clinicians to prevent the transfer of apparently normal yet abnormal embryos, thereby reducing the risks of unfavorable outcomes for patients.

## Introduction

Preimplantation genetic testing (PGT) can be applied in ART to analyze the DNA from oocytes (polar bodies) or embryo biopsies (cleavage stage or blastocyst) to determine genetic abnormalities. It includes testing for aneuploidy (PGT-A), monogenic/single gene defects (PGT-M), and chromosomal structural rearrangements (PGT-SR; Zegers-Hochschild *et al.*, 2017). PGT-A, PGT-M, and PGT-SR each interrogate fundamentally different types of genomic variants. PGT-A is the most widely implemented form of PGT, as it enables screening for chromosomal aneuploidies and the selection of euploid embryos. This is important, given that the incidence of chromosomal aneuploidies in oocytes increases with maternal age, and a large proportion of miscarriages and congenital disorders are ascribed to these chromosomal anomalies (Wapner *et al.*, 2012; Franasiak *et al.*, 2014; Demko *et al.*, 2016). PGT-M is the second most common form of PGT, aiming to discriminate risk alleles from the wild type alleles, preventing the transmission of inheritable disorders to the offspring in an autosomal dominant, autosomal recessive, or X-linked manner. Previous PGT-M methods have required the development of familial- and locus-specific assays tailored to each genetic condition and variant, which increased the time-to-pregnancy for the patients. PGT-SR is typically performed using standard PGT-A platforms to detect segmental gains or losses in chromosomal regions derived from unbalanced rearrangements. Nonetheless, such methods cannot distinguish balanced carriers from balanced non-carriers. As such, the offspring may encounter similar fertility issues when they reach reproductive age, including reduced fecundity, increased risk of miscarriage, and having affected children. Currently, other genomic abnormalities, including triploidy and uniparental diploidy, are under-appreciated by general PGT practice (Girardi *et al.*, 2024).

Polyploidy, primarily triploidy, has a pooled prevalence of 18.8% in early pregnancy losses and is the second most frequent abnormality as reported in a large cohort consisting of 24 900 miscarriages (Zhang *et al.*, 2018; Finley *et al.*, 2022). Approximately 98–99% of triploid conceptuses end in early miscarriage, with very few surviving to term or even after birth (Zaragoza *et al.*, 2000; Takabachi *et al.*, 2008). Triploid fetuses are associated with gross morphologic defects, including lethal neural tube defects and microcephaly (Philipp *et al.*, 2004). Moreover, triploidy has been associated with maternal conditions, such as gestational trophoblastic disease, preeclampsia, and hyperthyroidism, with the phenotypes and severity being influenced by the parental origin of the extra set of haploid chromosomes (Massalska *et al.*, 2020, 2021; Tidy *et al*., 2021). Currently, the morphological pronuclei check during early embryo development does not rule out all incidence of triploidy, especially diandric triploidy generated from a normal oocyte fertilized by a single diploid sperm when only two pronuclei are visualized (Rosenbusch, 2008). This was also accentuated by a large retrospective dataset collected from 1991 to 2018 by the Human Fertility and Embryology Authority (HFEA), which revealed that the incidence of molar pregnancy after fresh ICSI was 0.025% (27/107 571) (Nickkho-Amiry *et al.*, 2019). Considering the severity of triploidy and the fact that the IVF procedure co-cultures an oocyte and several good-quality sperm, which might increase dispermy fertilization, it is essential to accurately detect triploidy alongside other genetic abnormalities in PGT.

Another abnormal event, uniparental disomy (UPD), is copy-number neutral but characterized by a diploid copy number of specific homologous chromosomes deriving from only one parent with no copy from the other parent (Benn, 2021). UPD affecting single or multiple chromosomes was estimated to occur in 0.06% of human blastocysts by single nucleotide polymorphism (SNP) array-based comprehensive chromosome screening (CCS) and in 0.2–0.3% of newborns by whole-exome sequencing (Gueye *et al.*, 2014; Yauy *et al.*, 2020; Scuffins *et al.*, 2021). A more severe form of UPD, genome-wide UPD (gwUPD), is characteristic of a complete hydatidiform mole. In a large sporadic miscarriage cohort, gwUPD had a prevalence of 0.8% (196/24 900), among which 89 cases had a documented clinical or pathologic diagnosis of molar pregnancy (Finley *et al.*, 2022). Those surviving individuals were all females with mixoploidy and presented disorders of varying severity, including malignancy (Borgulová *et al.*, 2018). UPD, a key mechanism leading to absence of heterozygosity (AOH), presents as either isodisomy (two identical copies of one parental homolog, UPiD) or heterodisomy (both homologs from one parent, UPhD). The pathogenesis is mediated by imprinting defects and by homozygosity-induced autosomal recessive disease. As such, UPD or gwUPD can lead to early miscarriage if localized to chromosomes with imprinting genes controlling embryogenesis or fetal development (Lalou *et al.*, 2019). In addition, it will interfere with the interpretation of PGT-M as it masks the genotype identification by activating recessive alleles.

These challenging clinical concerns highlight the necessity of a comprehensive platform capable of detecting abnormal ploidy and heterozygosity status at the preimplantation level in order to optimize embryo selection and reduce unfavorable reproductive outcomes. To date, several concurrent PGT platforms have been developed (Yan *et al.*, 2015; Zamani Esteki *et al.*, 2015; Minasi *et al.*, 2017; Backenroth *et al.*, 2019; Ding *et al.*, 2020; Chen *et al.*, 2021; De Witte *et al.*, 2022; Masset *et al.*, 2022; Caroselli *et al.*, 2023); however, these studies have not provided validation data regarding genome-wide abnormalities, such as triploidy and gwUPD. Therefore, the objective of this study was to establish and validate a concurrent PGT platform and to investigate the incidence of gwUPD and triploidy in preimplantation embryos.

## Materials and methods

### Ethical approval

This study was approved by the Joint Chinese University of Hong Kong-New Territories East Cluster Clinical Research Ethics Committee (CREC-2010.432) and the Council on Human Reproductive Technology of Hong Kong (Research license R3005). Written informed consent was obtained from all participants for the retrieval of clinical information, storage of specimens, and analysis of genetic data. The study was carried out in compliance with the Helsinki Declaration.

### Study design

**Figure 1.**
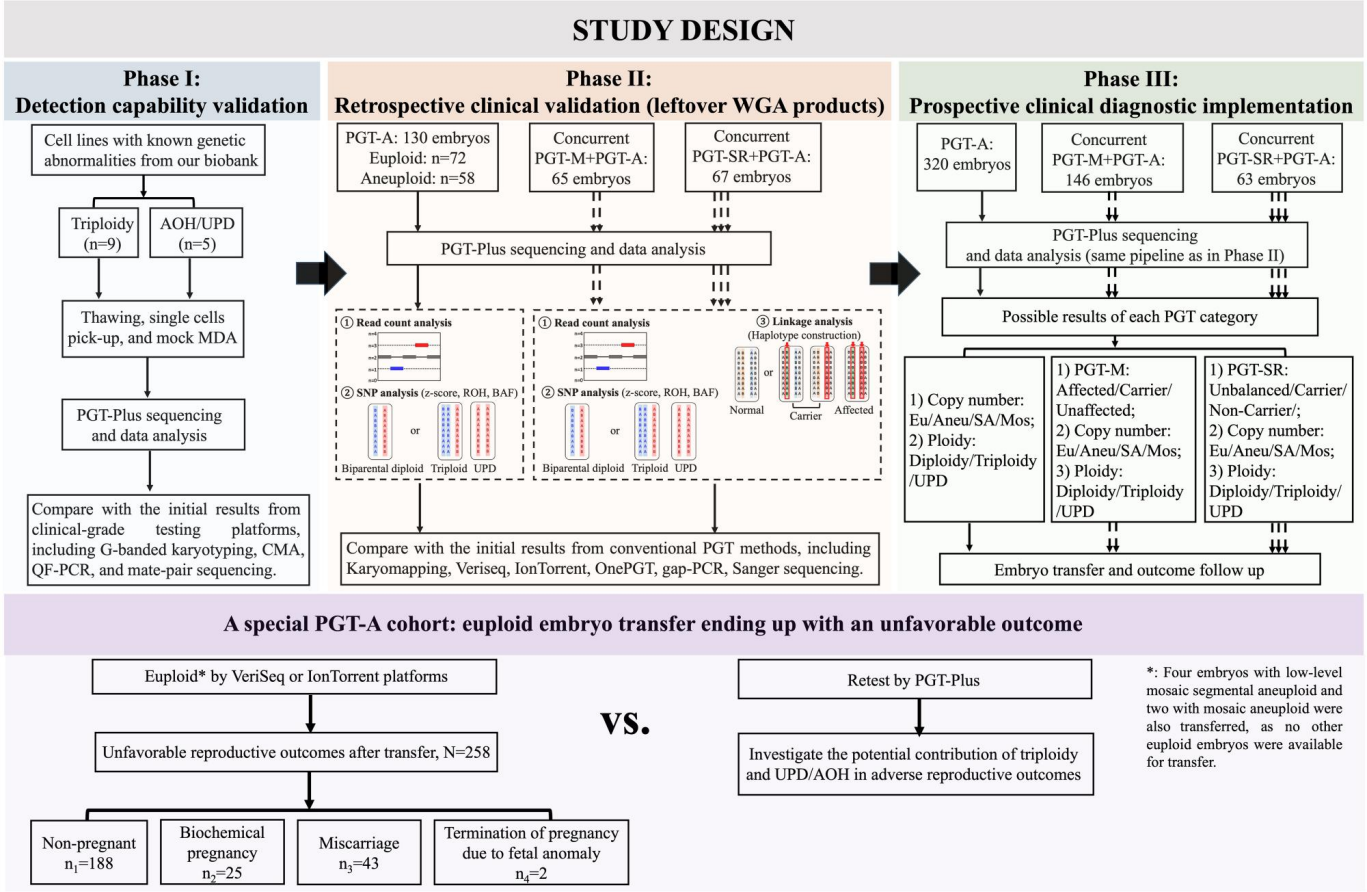
**Study design and workflow.** This study is structured in three phases. Phase I: validation of PGT-Plus using cell lines with ascertained genetic abnormalities. Phase II: blinded re-testing of archived leftover WGA samples from various PGT categories (PGT-A, M, SR) to benchmark PGT-Plus against conventional PGT platforms. Phase III: adopting the same data analysis pipeline as in Phase II, but integrating canonical read-count-based copy number analysis, SNP analysis, and linkage analysis. The synergistic results of each PGT category are shown in a separate box. AOH, absence of heterozygosity; UPD, uniparental disomy; MDA, multiple displacement amplification; WGA, whole-genome amplification; PGT-A/M/SR, preimplantation genetic testing for aneuploidies/monogenic disorders/structural rearrangements; SNP, single nucleotide polymorphism; ROH, runs of homozygosity; BAF, b allele frequency; Eu, euploidy; Aneu, aneuploidy; SA, segmental aneuploidy; Mos, mosaicism; QF-PCR, quantitative fluorescence PCR; CMA, chromosomal microarray.

The study design and workflow are illustrated in Fig. 1.

This study was composed of three phases. Phase I validated the detection capability of PGT-Plus for UPD/AOH and triploidy in cell lines with ascertained genetic abnormalities from the biobank of the Prenatal Genetic Diagnosis Laboratory at the Chinese University of Hong Kong (PGD Lab, CUHK). Phase II performed PGT-Plus on archived WGA products from various PGT cycles between January 2019 and July 2023 at Prince of Wales Hospital, including PGT-A, PGT-M, and PGT-SR. PGT results from previous platforms were blinded during the PGT-Plus data analysis. Phase III applied the PGT-Plus platform prospectively for clinical implementation within a single PGT laboratory from August 2023 to June 2024.

Specifically, an additional unique cohort, with adverse reproductive outcomes after embryo transfer, was retested to identify any triploidy and UPD among these embryos previously classed as euploid by conventional PGT-A platforms. The unfavorable outcomes include non-pregnant, biochemical pregnancy, and miscarriage.

### Cell lines and treatment

Multiple cell lines with triploidy, UPD, or AOH from the biobank of the Prenatal Genetic Diagnostic Laboratory, The Chinese University of Hong Kong (PGD Lab, CUHK), were used to evaluate the PGT-Plus platform detection capability. These samples were previously tested using at least one of the following methods: G-banded karyotyping, quantitative fluorescence PCR (QF-PCR), chromosomal microarray, and mate-pair sequencing (Dong *et al.*, 2019, 2021; Qian *et al.*, 2025). The details are shown in Supplementary Table S1.

Four- to eight-cell aliquots of each cell line were picked under an inverted microscope and transferred into a 0.2 ml PCR tube containing 4 μl of phosphate-buffered saline. Whole-genome amplification (WGA) was performed using the multiple displacement amplification (MDA) method with the REPLI-g Single Cell Kit (Qiagen, Hilden, Germany) as per the manufacturer’s instructions to simulate the genetic material obtainable from trophectoderm biopsies.

### DNA extraction, WGA, library preparation, and sequencing

Genomic DNA (gDNA) was extracted from the peripheral blood of the parental and reference family members, if any, using the commercial DNeasy Blood & Tissue Kit (Qiagen). WGA was performed on embryo biopsies using one of the following two methods, preferentially selected based on the specific PGT purpose, although both were compatible with the PGT-Plus approach. For PGT-M, MDA was employed using the REPLI-g Single-Cell Kit (Qiagen) to ensure high fidelity for single-gene disorder analysis. For PGT-A, a PCR-based method, PicoPLEX (Takara, San Jose, CA, USA) was used due to its superior uniformity in genome coverage, which is critical for copy number variant (CNV) detection. In cases of PGT-SR, the WGA method was chosen based on the diagnostic goal. PicoPLEX was used to identify genomic dosage imbalances, while MDA was applied when the additional detection of balanced structural rearrangement carriers was required.

All gDNA and WGA products were subsequently used for library preparation. The input amount varied by DNA types: 200 ng was required for MDA products, while 400 ng was used for PicoPLEX products and gDNA. DNA was enzymatically fragmented with restriction endonucleases I and II in a thermal cycler program set to 37°C for 15 min, 65°C for 20 min, 4°C for 1 min. Following fragmentation, adapters were added to DNA fragments and heated to 65°C to increase adapter–fragment interactions and decrease fragment–fragment interactions. Adapters were then ligated to DNA fragments using DNA ligase, under reaction conditions of 22°C for 15 min, 65°C for 10 min, 4°C for 1 min, followed by a hold at 4°C. Further, to remove excess unligated adapters and dimer artifacts, ligation products were purified using AMPureXP magnetic beads (Beckman Coulter Genomics, Danvers, MA, USA). A subsequent size selection by the same beads was performed to isolate adapter-tagged DNA fragments with a target size range of 200–400 bp. A final PCR amplification was performed to generate uniquely barcoded libraries. Libraries were quantified by the Qubit dsDNA HS assay (Thermo Fisher, Waltham, MA, USA), and those with a concentration exceeding 1.6 ng/μl were considered to have passed quality control.

Qualified libraries were pooled in equal molarity and sequenced on the MGISEQ-2000 platform (MGI, Shenzhen, China) to a minimum depth of 80 million paired-end reads (2×100 bp read length) per library as previously described (Cao *et al.*, 2022; Chau *et al.*, 2022). Detailed sequencing data analysis pipelines are provided in the Supplementary Materials and methods.

### Conventional gold standard PGT methods for diagnosis consistency comparison

To evaluate the performance of PGT-Plus for relative haplotype phasing and copy-number analysis, Karyomapping/Asian Screening Array (Vitrolife, Gothenburg, Sweden) and OnePGT (Agilent, Santa Clara, CA, USA) platforms were performed in parallel for all blastocyst biopsies in Phase II, using BlueFuse Multi software (Vitrolife) and Alissa system for data analysis and visualization, respectively (Masset *et al*., 2019). A third reference method, including short-tandem repeat analysis, gap-PCR combined with Sanger sequencing, was also utilized where applicable, especially for Alpha Thalassemia due to Southeast Asian-type large deletions.

## Results

### Phase I: detection capability of PGT-Plus for UPD/AOH and triploidy in cell lines with ascertained genetic abnormalities

Fourteen samples from cryopreserved cell lines with previously ascertained triploidy and UPD/AOH were included for technical validation (Supplementary Table S1). Of these, four had parental samples, confirming the parental origin in agreement with prior results. Nine were triploid, and five were AOH samples with descending size, ranging from whole genome-wide to ∼5 Mb. All samples showed 100% concordance with the original test results. The results of the comparison between various methods are provided for one representative case of maternally originated triploidy (Supplementary Fig. S1).

### Phase II: clinical validation of PGT-Plus in a retrospective PGT cohort

With the high level of concordance established in cell lines, further validation on a retrospective PGT cohort (n = 262 embryos) was performed using archived WGA samples. This cohort comprised of 65 embryos from 8 cycles for concurrent PGT-M and PGT-A, 67 embryos from 9 cycles for concurrent PGT-SR and PGT-A, and 130 embryos for PGT-A. Triploidy and gwUPD embryos accounted for 0.8% (2/262) and 1.9% (5/262) in this cohort, respectively (Table 1).

**Table 1.** Triploidy and uniparental disomy in various sample cohorts.

	PGT category	Maternal/paternal age (means)	Sample size		Triploidy		Uniparental disomy	
			No. of embryos	No. of cycles	No. of embryos	No. of cycles	No. of embryos	No. of cycles
**Phase II: retrospective clinical validation**	**PGT-A**	37.7/40.0	130	66	2	2	2 (gwUPD)	2
	**PGT-M + A**	34.4/37.0	65	8	0	0	2 (gwUPD, mat)	2
	**PGT-SR + A**	33.8/37.9	67	9	0	0	1 (gwUPD, mat)	1
**Subtotal-1**		37.0/39.5	262	83	2 (0.8%)	2 (2.4%)	5 (1.9%)	5 (6.0%)
**Phase III: prospective diagnostic implementation**	**PGT-A**	37.8/40.0	320	84	2	2	1 (UPD18)	1
	**PGT-M + A**	33.7/37.5	146	28	1	1	2 (gwUPD, mat)	2
	**PGT-SR + A**	33.7/36.7	63	11	0	0	1 (gwUPD, mat)	1
**Subtotal-2**		36.5/39.1	529	123	3 (0.6%)	3 (2.4%)	4 (0.8%)	4 (3.3%)
**A special cohort: euploid embryos transferred with adverse outcomes** ^a^		37.0/39.2	258	202	3 (1.2%)	3 (1.5%)	6 (2.3%, gwUPD)	6 (3.0%)
**Total**		36.8/39.2	1049	408	8 (0.8%)	8 (2.0%)	15 (1.4%)	15 (3.7%)

a Includes not being pregnant, biochemical pregnancy, and miscarriage.

gwUPD, genome-wide uniparental disomy; mat, maternal origin; PGT-A/M/SR, preimplantation genetic testing for aneuploidy/monogenic disorder/structural rearrangement.

In embryos for PGT-A alone, random selection was applied, among which 72 were euploid and 58 were abnormal based on prior PGT platforms. The mean maternal and paternal age was 37.7 and 40.0 years old, respectively. PGT-Plus revealed previously unknown gwUPD in two euploid embryos (23H004-4 and 23H027-4), which would alter clinical decisions. Among abnormal embryos, aneuploidy and segmental aneuploidy covering all chromosomes, either in mosaic or non-mosaic forms, were included. Two embryos showing simple copy-number abnormalities by the prior PGT-A approach were revealed to be triploidy by PGT-Plus, including a case of 69,XXX and a case of 69,XXY. All remaining embryos showed concordance between platforms.

**Figure 2.**
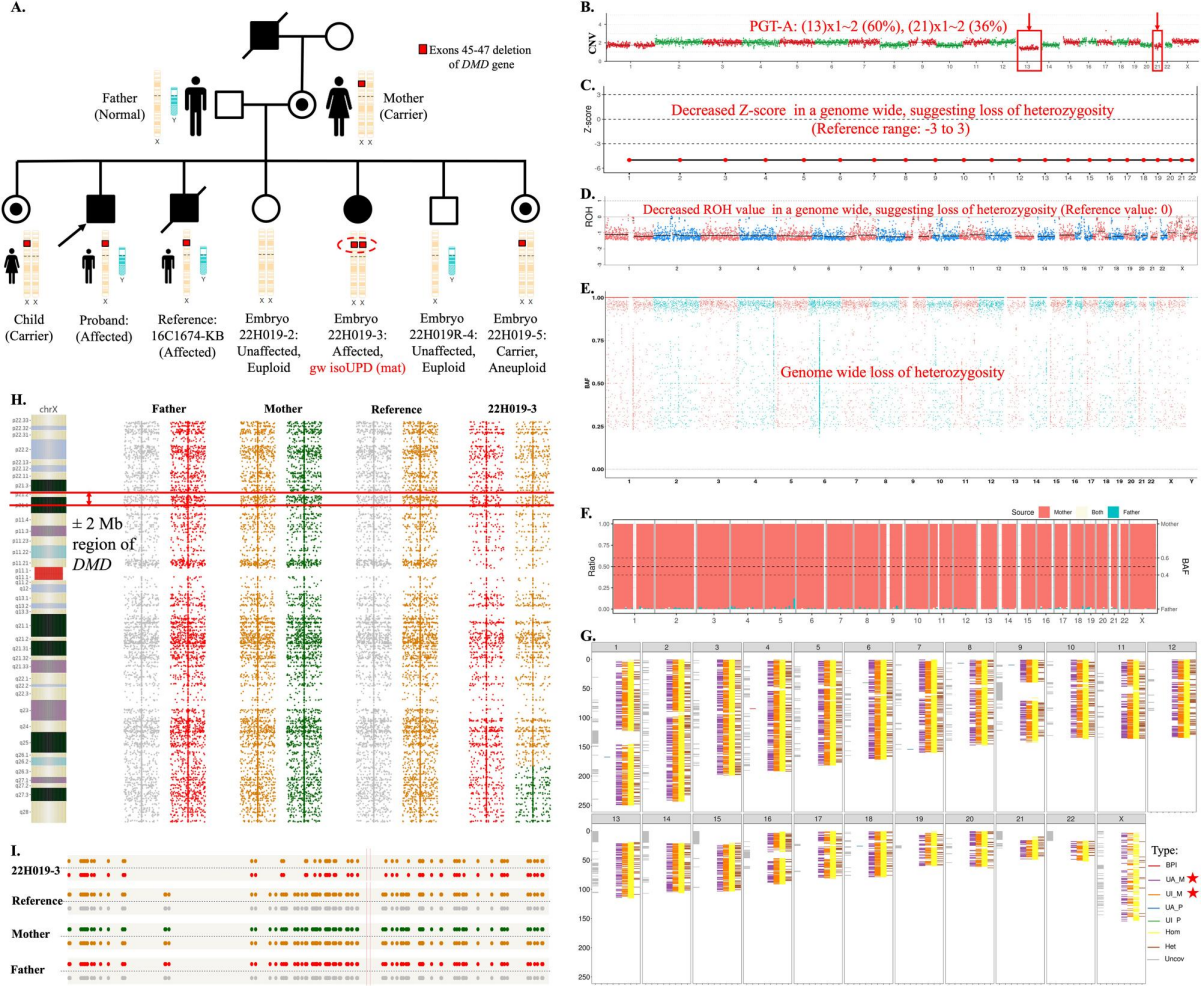
**Genome-wide maternal isodisomy adds to the complexity of PGT-M: a case illustration of embryo 22H019-3 from a family with Duchenne Muscular Dystrophy.** (**A**) Pedigree. (**B**) PGT-A analysis: CNV analysis indicates a female embryo with mosaic aneuploidy. (**C**–**E**) Ploidy analysis: genome-wide alterations collectively indicated genome-wide loss of heterozygosity, consistent with gwUPD, as evidenced by Z-score values <–3 (C), ROH values <–1 (D), and BAF values deviating from 0.5 (E). (**F** and **G**) Parental origin analysis of gwUPD: SNP analysis revealed that gwUPD in embryo 22H019-3 was of maternal origin, characterized by the presence of maternal genome and absence of paternal contribution. (**H** and **I**) Haplotyping: relative phasing demonstrated that embryo 22H019-3 inherited the mutant maternal haplotype (in orange). In the context of coexisting maternal genome-wide isodisomy, this embryo is thus affected by this *DMD* condition. DMD, Duchenne Muscular Dystrophy; gwUPD, genome-wide uniparental disomy; isoUPD, uniparental isodisomy; ROH, runs of homozygosity; BAF, b-allele frequency; SNP, single-nucleotide polymorphism; Uncov, uncovered; BPI, both parental inheritance; UA_M/P, maternally/paternally inherited SNPs but do not allow distinction between isodisomy and heterodisomy; UI_M/P, SNPs consistent with maternal/paternal isodisomy; Hom, homozygosity; Het, heterozygosity.

**Figure 3.**
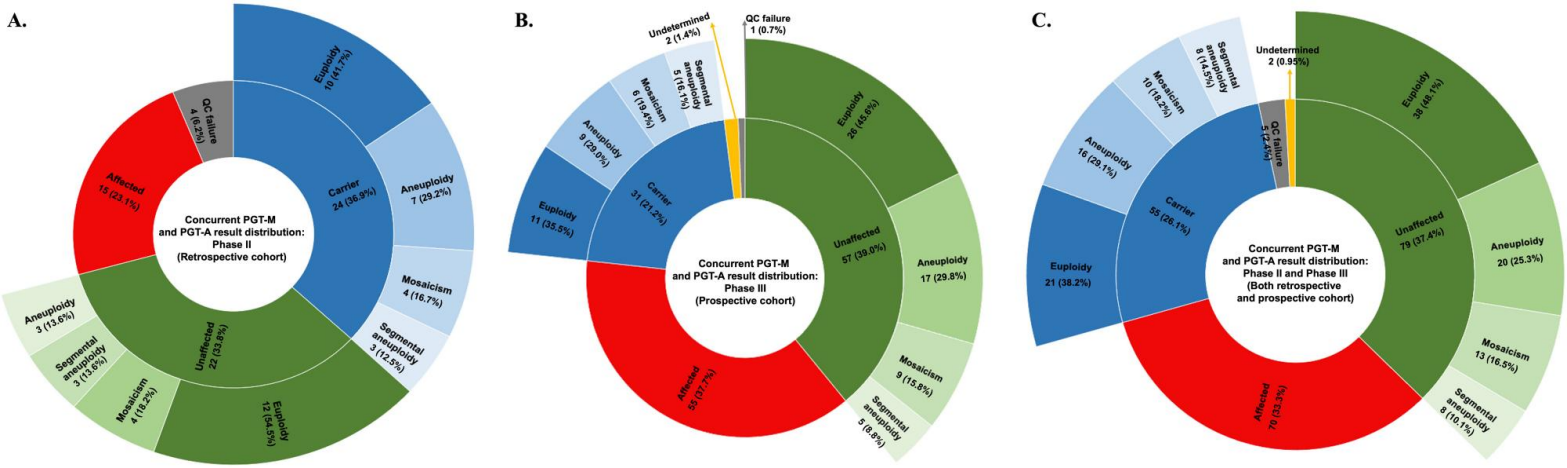
**Proportion of various copy-number results distribution among each linkage-analysis classification in embryos performing concurrent PGT-M and PGT-A.** (**A**) Results from the retrospective cohort of Phase II: the inner circle depicts the various results of PGT-M linkage analysis: 36.9% (24/65) were carriers, 33.8% (22/65) were unaffected, 23.1% (15/65) were affected, and 6.2% (4/65) failed QC due to poor biopsy quality. The outer circle depicted the various results of PGT-A copy-number analysis among those ‘transferrable’ ones (carrier and unaffected) by PGT-M analysis. The combined PGT-M and PGT-A assessment revealed that only 22 of the 65 embryos (33.8%) were eligible for transfer. (**B**) Results from the prospective cohort of Phase III: as shown in the inner circle, 21.2% (31/146) were carriers, 39.0% (57/146) were unaffected, 37.7% (55/146) were affected, and 0.7% (1/146) failed QC. Particularly, the phase of two embryos was undetermined due to biological gwUPD. A minority of embryos, 25.3% (37/146), met the criteria for transfer after undergoing both PGT-M and PGT-A analysis. (**C**) Cumulative PGT-M and PGT-A analysis from both phases: according to this sunburst graph, the combined analysis of embryos from the retrospective and prospective cohorts indicated that only 28.0% (59/211) met the criteria for transfer after concurrent PGT-M and PGT-A. PGT-M, preimplantation genetic testing for monogenic disorders; PGT-A, preimplantation genetic testing for aneuploidy; gwUPD, genome-wide uniparental disomy.

A total of 65 embryos from 8 cycles undergoing concurrent PGT-M and PGT-A were included, with an average maternal age of 34.4 and paternal age of 37.0. Diseases with various modes of inheritance, including autosomal recessive, autosomal dominant, and X-linked recessive, were included (Supplementary Table S2). All phasing results and copy-number profiles were consistent between PGT-Plus and other reference platforms. Nonetheless, genome-wide uniparental isodisomy was revealed in two embryos (22H019-3 and 21H244-9). Previous PGT results for embryo 22H019-3 showed a mosaic aneuploid female embryo carrying the maternally inherited pathogenic Duchenne Muscular Dystrophy variant (Supplementary Fig. S2). However, the maternal genome-wide isoUPD not only was homozygous for the variant, but also is associated with an increased risk of becoming a hydatidiform mole, exemplifying the necessity of detecting UPD in preimplantation embryos (Fig. 2 and Supplementary Fig. S3). Following the transfer of the unaffected and euploid embryo (22H019-2), the patient delivered two healthy newborns, who were monochorionic diamniotic twins. In addition, for another family affected by the *ABCD1* mutation, embryo 21H244-9 was unaffected and euploid by the initial platform and would have been prioritized for transfer previously if the gwUPD information had not been revealed by PGT-Plus. Furthermore, among all these 65 embryos, 70.7% (46/65) were unaffected, or carriers of the parental monogenic mutation, but only 47.8% (22/46) were euploid and transferable (Fig. 3A).

**Figure 4.**
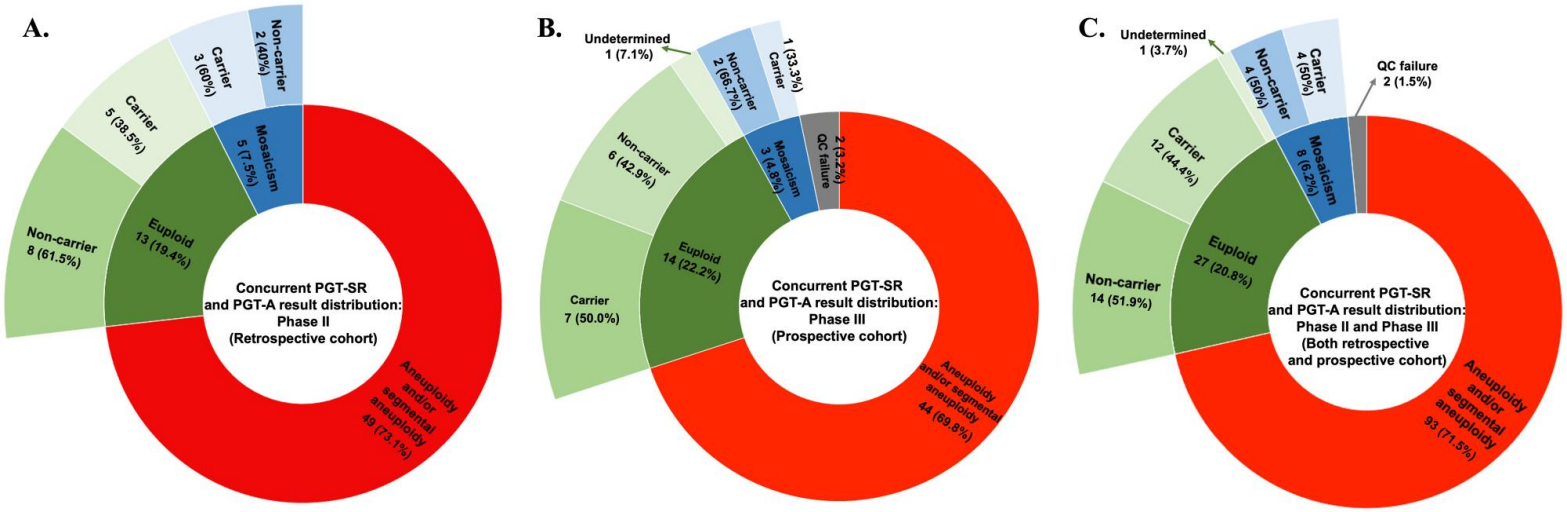
**Distribution of copy-number findings across linkage-analysis classifications in embryos undergoing concurrent PGT-SR and PGT-A.** (**A**) Results from the retrospective cohort of Phase II: as shown in the inner circle, only 19.4% of these embryos were euploid (13/67) by PGT-A analysis. PGT-SR linkage analysis, represented by the outer circle, revealed that a substantial proportion (39.5%, 5/13) of these euploid embryos were translocation carriers. (**B**) Results from the prospective cohort of Phase III: only half of the euploid embryos (7/14) were translocation carriers. Particularly, one embryo had an undetermined phase due to isoUPD on the affected chromosome. (**C**) Summary of concurrent PGT-SR and PGT-A from both cohorts: combined analysis of both cohorts revealed that only 10.8% (14/130) of embryos undergoing PGT-SR and PGT-A were balanced non-carriers, representing the subset with the potential for a pregnancy free of the familial translocation. PGT-SR, preimplantation genetic testing for structural rearrangements; PGT-A, preimplantation genetic testing for aneuploidy.

For embryos undergoing concurrent PGT-SR and PGT-A, 29 were from families carrying a Robertsonian translocation, and 38 were from families carrying a reciprocal translocation (Supplementary Table S3). The mean maternal and paternal ages were 33.8 and 37.9. Among these embryos, only 19.4% (13/67) were euploid, which is expected given the increased probability of gamete segregation imbalance caused by parental translocation. Notably, 38.5% (5/13) of these euploid embryos were identified as balanced translocation carriers (Fig. 4A). In addition, one mosaic segmental aneuploid embryo (embryo ID: 21H193-3) was revealed to be gwUPD. Without these gwUPD results, embryos with mosaic abnormalities may be considered for transfer and lead to an unfavorable pregnancy outcome.

### Phase III: diagnostic clinical implementation in a prospective cohort

Based on the robust validation results of this method in the previous two phases, PGT-Plus was implemented in a prospective clinical cohort with indications on all three categories of PGT. A total of 529 embryos were tested (mean maternal age: 36.5 years old; mean paternal age: 39.1 years old). Among them, 146 embryos were from couples with monogenic disorders, and 63 were from couples with reciprocal or Robertsonian translocations. The mean maternal age was 33.7 years old for both groups, while the mean paternal ages were 37.5 and 36.7, respectively. The family and disease information is provided in Supplementary Tables S2 and S3, respectively. The remaining 320 embryos were indicated for PGT-A only (mean maternal age: 37.8 years old; mean paternal age: 40.0 years old).

Concurrent PGT-M and PGT-A analysis included 146 embryos from 28 cycles of 22 couples, involving 9 autosomal dominant conditions, 9 autosomal recessive conditions, 2 X-linked conditions, and 2 microdeletion syndromes. According to phasing analysis, 89 embryos were unaffected by the parental monogenic disorder or carrier. Among these embryos, only 42.7% (38/89) were euploid, 26 were aneuploid, 10 had a CNV larger than 4 Mb, which was classified as likely pathogenic and pathogenic according to the American College of Medical Genetics and Genomics (ACMG) guideline, and the remaining 15 embryos showed mosaicism (Fig. 3B). Notably, one embryo 23H116-5 from a family with autosomal dominant polycystic kidney disease showed genome-wide maternal uniparental isodisomy, despite being euploid, which interfered with phasing of the paternal variant-containing haplotype. However, the Sanger sequencing result for this embryo showed it to be wild type, suggesting that this embryo would be transferable if the PGT-Plus platform had not been applied. Moreover, embryo 23H020-4 was unaffected by the parental 16p11.2 microdeletion (614 kb in size) syndrome but was triploid in addition to mosaic monosomy 16.

**Figure 5.**
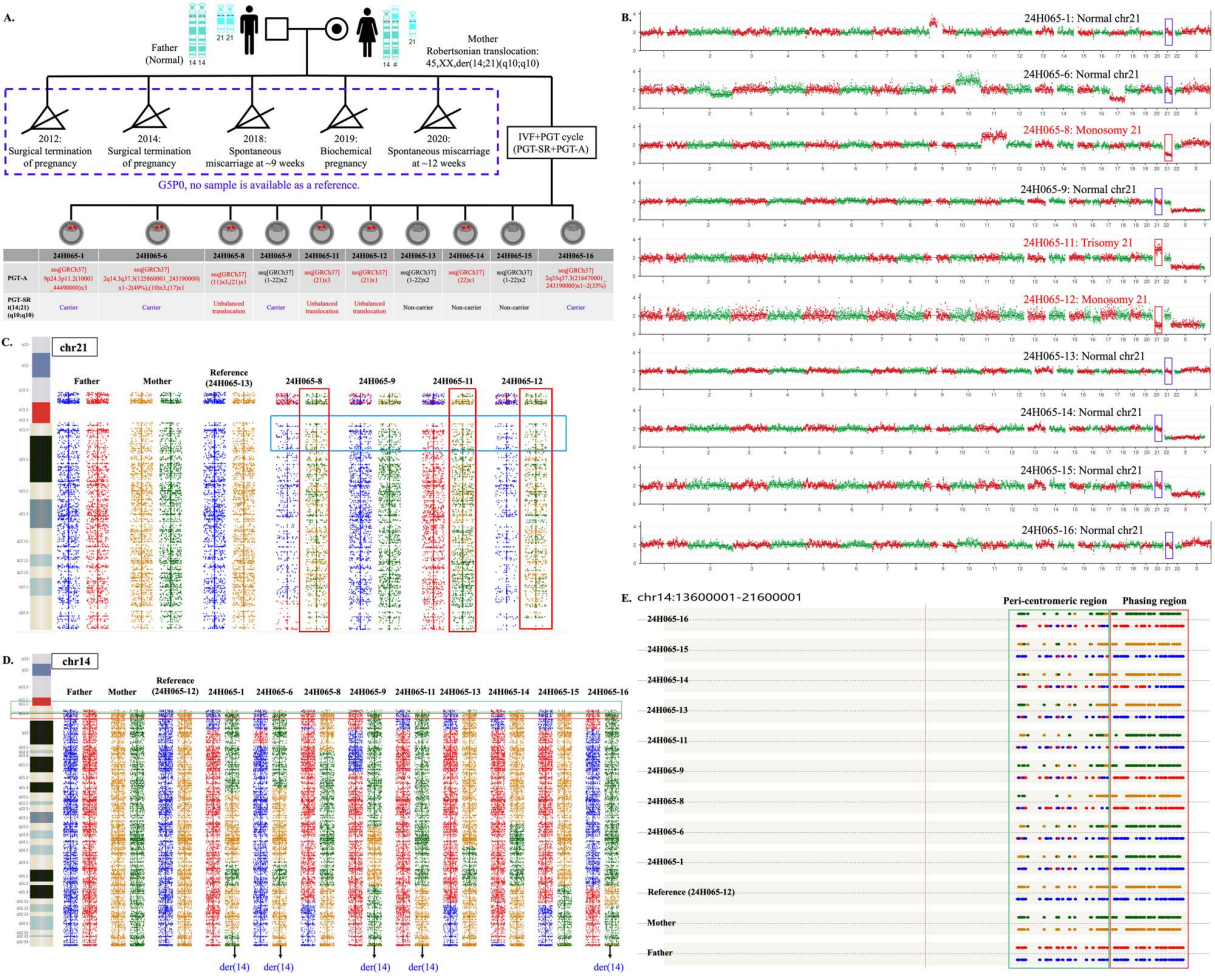
**PGT-Plus enabled identification of translocation carriers in reference-free families.** (**A**) Pedigree: rob(14; 21)(q10; q10) family. (**B**) PGT-A results: embryos #8 and #12 had monosomy 21, while #11 had trisomy 21 (red rectangle). No other embryos showed aneuploidy involving chr14 or chr21. (**C**) Identification of aneuploidy etiology on chr21: mixed maternal haplotypes (yellow and green dots) were observed in the 2 Mb region downstream of the 21q10 breakpoint (blue rectangle) in embryos #8, #11, and #12, consistent with a meiotic segregation error caused by maternal Robertsonian translocation. (**D** and **E**) SNP phasing to distinguish carriers from non-carriers: using the unbalanced embryo #12 as the reference, embryos #1, #6, #9, #11, and #16 were revealed to carry derivative chr14, while all others inherited the normal chr14 from the mother. All informative SNPs supported the assigned phases. Combined with PGT-A results, this approach enabled the discrimination between carriers and non-carriers among balanced embryos. Der, derivative; G5P0, five gestation and zero parity; chr, chromosome; SNP, single nucleotide polymorphism; PGT-A, preimplantation genetic testing for aneuploidy.

There were 63 embryos from 8 families who underwent concurrent PGT-SR and PGT-A, with 18 from families carrying Robertsonian translocation and 45 from families carrying reciprocal translocation. Among them, only 22.2% (14/63) were euploid, while half of them were balanced carriers (Fig. 4B). In addition, one embryo had an undetermined phase because it was genome-wide isoUPD. Two samples failed QC due to the compromised quality of WGA products. In particular, PGT-Plus enabled identification of translocation carriers even in families lacking a reference sample. We illustrate this using a case of Robertsonian translocation in which the female partner carried a 45,XX,der(14;21)(q10;q10) karyotype. The couple had experienced five failed pregnancies, with no retained biological samples for use as a reference (Fig. 5A). The first step was to determine the etiology of aneuploidy involving chromosomes 14 and 21 in the embryos (Fig. 5B), specifically, whether it resulted from parental translocation or random maternal meiosis II error, and then to select an embryo with a genomic dosage imbalance derived from the parental translocation to serve as the reference. To achieve this, embryo #13, which was disomic for chromosome 21, was used as a reference. Three aneuploid embryos (#8, #11, and #12) exhibited mixed maternal haplotypes in the 2 Mb region downstream of the breakpoint at 21q10, indicating a meiotic segregation error attributable to the maternal Robertsonian translocation (Fig. 5C). In contrast, chromosome 21 aneuploidy resulting from a random maternal meiosis II error would manifest as a single maternal haplotype in this region due to non-disjunction of identical sister chromatids. Accordingly, the aneuploidies involving chromosome 21 in embryos #8, #11, and #12 were all attributable to the maternal Robertsonian translocation, and these embryos could all serve as references for subsequent haplotyping analysis (Fig. 5D and E).

Of the 320 PGT-A embryos, one of them was detected with UPD 18. In addition, two embryos were triploid. All others were diploid, and the genetic abnormality distribution profile was comparable to previous reports.

Furthermore, clinical outcomes for embryo transfer completed before November 2025 were followed up (Supplementary Fig. S4). The transferred embryos included 70 from PGT-A, 16 from PGT-M+A, and 8 from PGT-SR + A cycles. The aggregated Phase III outcomes demonstrated an implantation rate of 61.7% (58/94), a clinical pregnancy rate of 58.9% (53/94), and a miscarriage rate of 7.8% (7/94). Notably, the observed miscarriages were exclusive to the PGT-A cohort, a known poorer-prognosis group characterized by the highest average maternal age (37.8 years), suggesting an influence of maternal rather than embryonic factors in these pregnancy losses.

### Triploidy and gwUPD in a cohort with unfavorable outcomes after single embryo transfer determined by prior PGT-A platform

During the study period, 258 blastocysts classified as transferable (euploid or mosaic segmental aneuploid) by prior PGT-A platforms were transferred to patients (mean maternal age: 37 years old; mean paternal age: 39.2 years old). These transfers resulted in adverse reproductive outcomes, including 188 not being pregnant, 25 biochemical pregnancies, 43 early miscarriages, and 2 terminations of pregnancy due to fetal anomalies. Re-testing by PGT-Plus revealed that a subset of these embryos harbored genome-wide abnormalities, although they were copy-number neutral, with 1.2% (3/258) being triploid and 2.3% (6/258) being gwUPD. These abnormalities, if detected previously, would have prohibited transfers.

### Determination of parental origin and stage of nondisjunction in preimplantation embryos with genome-wide abnormalities

**Figure 6.**
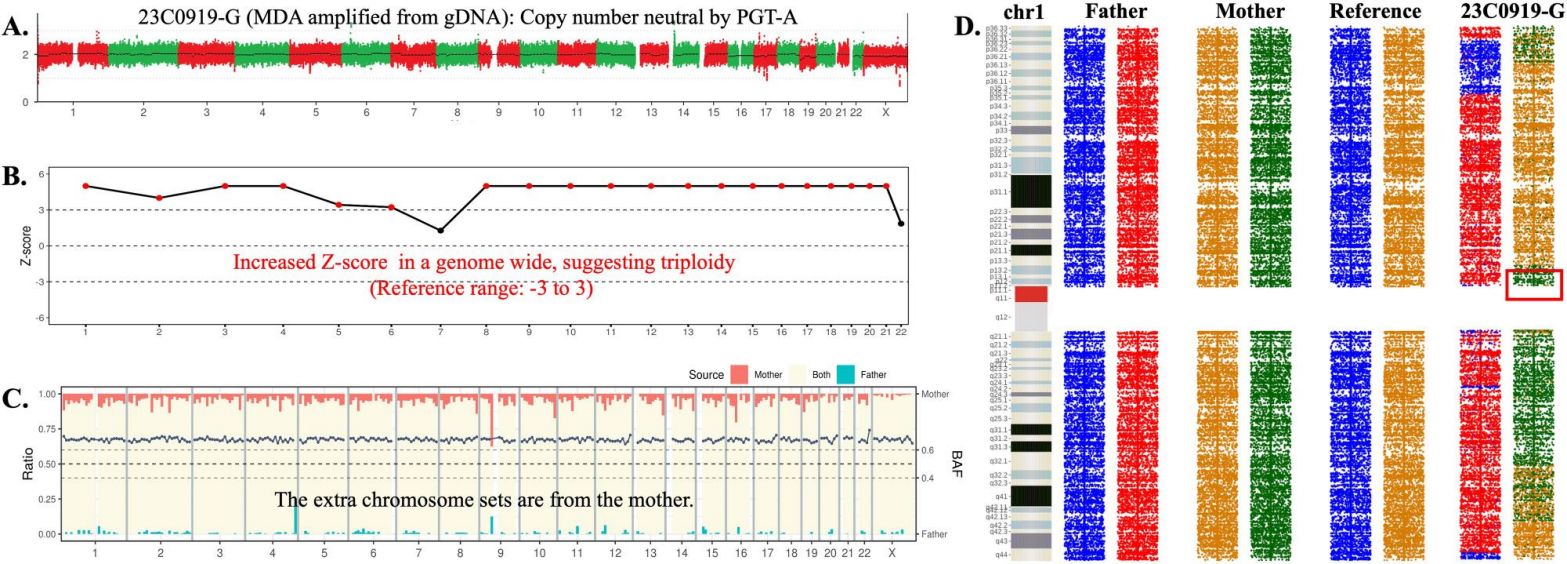
**Determination of parental origin and stage of nondisjunction in triploid embryos.** (**A**) Copy number distribution: PGT-A analysis revealed a copy-number neutral profile, resembling that of a ‘euploid’ female. The gDNA sample was amplified by MDA, resulting in scattered sequencing windows. (**B**) Z-score: genome-wide elevation of Z-score values was suggestive of triploidy of this sample. (**C**) Parental origin analysis of triploidy: the curved lines of all chromosomes skewed toward the maternal side, suggesting excessive maternal genome contribution. This pattern is consistent with digynic triploidy, in which the extra chromosome set is of maternal origin. The maternal and paternal genomes are shown in red and blue, respectively. (**D**) Stage of nondisjunction analysis of triploidy: the presence of only one maternal haplotype near the centromere (shown for chromosome 1 as an example) is characteristic of maternal meiosis II error (red rectangle). In such an error, the failure of sister chromatid separation results in the inheritance of two identical maternal haplotypes, while recombination events are confined to distal chromosome arms. Similar profiles were observed for all other chromosomes (data not shown). Collectively, these findings indicate that the digynic triploidy arose from a meiosis II error. gDNA, genomic DNA; MDA, multiple displacement amplification; chr, chromosome; PGT-A, preimplantation genetic testing for aneuploidy.

Parent samples were available for one triploid embryo and six embryos with gwUPD to determine the parental origin and stage of the nondisjunction event. This triploidy case was consistent with maternal meiosis II error (Fig. 6). Meanwhile, SNP-based phasing revealed that all six gwUPD cases were of maternal origin, characterized by the presence of the maternal genome and absence of paternal genetic contributions (Table 1). These findings were independently validated in all six mother–father–embryo trios using orthogonal QF-PCR with a panel of 26 short tandem repeat markers targeting chromosomes 13, 18, 21, X, and Y. SNPs supporting the maternal origin of a representative case are shown in Fig. 2G, with validation by QF-PCR provided in Supplementary Fig. S3 and Supplementary Table S4.

## Discussion

This study demonstrated the feasibility and validity of the PGT-Plus approach as a comprehensive concurrent PGT platform, allowing for the simultaneous detection of PGT-A, PGT-M, PGT-SR, triploidy, and UPD, as well as the identification of parental origin and stage of nondisjunction within a single assay. In addition to performance superiority of the detection scope, PGT-Plus is also advantageous in its cost-effectiveness and turnaround time. The use of the MGISEQ-2000 sequencer offers a lower per-sample cost compared to Illumina-based platforms. With a requirement of 80 million reads per sample for robust SNP analysis, a sequencing pool consisting of 32–36 samples can be loaded to one flowcell, irrespective of their PGT application (A, M, or SR) and DNA source (gDNA, MDA, or PicoPLEX WGA products), thereby eliminating the need for clinics with low PGT volumes to batch samples, which optimizes workflow efficiency and reduces both cost and turnaround time greatly (Supplementary Table S5).

In this study, for embryos undergoing PGT-M, 56.0% (75/134) of unaffected or carrier ones had numerical abnormalities, including aneuploidy, segmental aneuploidy, and mosaicism (Fig. 3C); thus, it was important to perform PGT-A concurrently with PGT-M. For PGT-SR, it additionally enabled the differentiation of balanced carriers from balanced noncarrier embryos, preventing the transmission of parental translocation to future generations in 45.7% (12/35) of the transferable embryos when only considering copy-number neutral embryos (Fig. 4C). This discrimination was unachievable by conventional PGT-A platforms. In addition, this study demonstrated the detection capacity of PGT-Plus in phasing cryptic translocations where the translocation segments were smaller than 5 Mb. These specific chromosomal rearrangements were recently detected by utilizing the single-tube long-fragment read whole-genome sequencing method (stLFR WGS), which significantly impacted clinical decisions for couples with cryptic balanced translocations and recurrent pregnancy loss (Jiang *et al.*, 2025). Taken together, these findings underscore the value of PGT-Plus in enabling synergistic and integrated detection of complex genetic conditions. Within this framework, CNV and b-allele frequency (BAF) data are interpreted jointly, as abnormal BAFs can be caused by CNVs as well. Moreover, the diploid status of the target locus is confirmed prior to haplotype phasing. This integrated analysis is particularly important to eliminate the interference of abnormal copy number on haplotype analysis of the gene of interest in PGT-M, such as the *SERPINA1* gene on chromosome 14 in the context of trisomy 14. In such scenarios, data from different angles, that is, read-depth coverage data for CNV analysis and SNP data for haplotyping analysis, cross-validate each other and provide mutual reinforcement. Therefore, for prospective parents undergoing PGT, PGT-Plus could be considered as a first-tier diagnostic approach, irrespective of the initial indication (A, M, or SR). This is justified by its expanded detection scope and analytical consistency while maintaining a favorable cost and time profile compared to conventional platforms.

Genome-wide anomalies, such as triploidy and gwUPD, typically occur from a heterogenic division event, which might be attributed to the failure of the parental genome uniting on a single spindle and the asynchrony of different pronuclei undergoing the cell cycle (Masset *et al.*, 2021; De Coster *et al.*, 2022). They may be more common than expected in human pregnancies and could well be the cause of a significant portion of IVF failures (Zhao *et al.*, 2025), but they are unrecognized by current PGT-A methods. Techniques applied in PGT have evolved over time, with next-generation sequencing (NGS) as the mainstay for PGT-A and SNP array for PGT-M. Although an imbalance in chromosome X and Y coverage may indicate certain triploid genotypes, such as 69,XXY, this pattern does not apply to haploidy or triploidy lacking a Y chromosome, such as 69,XXX (Mutia *et al.*, 2019). Consequently, haploid and triploid embryos are frequently misclassified as diploid when assessed solely by copy-number-based PGT-A. This diagnostic incompetence offers a plausible explanation for why some embryos classified as euploid may nonetheless result in miscarriage, as supported by our finding that 3.5% of seemingly ‘euploid’ embryos actually had genome-wide aberrations (gwUPD or triploidy). To further support this interpretation, a negative control cohort from embryos in Phases II and III of our study was assembled. Among a total of 163 embryos transferred, 80 resulted in successful pregnancies (live birth or ongoing pregnancy), and none of these embryos showed any ploidy abnormalities during pregnancy, prenatal diagnosis, or after birth. This finding was further corroborated by an independent review of our clinical laboratory database, which identified no instances of gwUPD or triploidy among embryo transfers leading to live birth or ongoing pregnancy. In addition, clinical records of patients in the special cohort were reviewed for maternal factors known to contribute to adverse pregnancy outcomes. No remarkable evidence of conditions such as adenomyosis, endometriosis, endocrine abnormalities, or autoimmune disorders was documented. These observations support the likelihood that the adverse outcomes observed are attributable to embryonic rather than maternal factors.

Triploidy had an incidence of 0.5% (89/18791) in blastocysts by a targeted NGS-based platform but 1.0% (188/19369) by the genome-wide single-nucleotide polymorphism microarray method reported by the same research team (Marin *et al.*, 2018; Kratka *et al.*, 2023). However, triploidy contributes 2% of human natural conceptions and 14% of cytogenetically abnormal pregnancies (Wang *et al.*, 2014). In this current study, 0.8% (8/1049) of preimplantation embryos were triploid. Detection of abnormal ploidy at the preimplantation level can avoid the transfer of embryos that lead to implantation failure, miscarriage, and potential for trophoblastic disease.

UPD is a well-known mechanism of genetic disease, and as such, the ACMG has published a guideline for prenatal and postnatal UPD testing (Del Gaudio *et al.*, 2020). Abnormal phenotypes will be caused if UPD affects chromosomes 6, 7, 11, 14, 15, or 20 due to parental-of-origin-dependent imprinting differences. In the extreme scenario where all chromosomes are involved (gwUPD), the clinical consequences are severe and encompass a broad spectrum of abnormalities. Even reported in mosaic form with mosaic levels ranging from 20% to 80%, all 16 prenatal and products-of-conception cases reported fetal/neonatal demise, multiple congenital anomalies, or abnormal ultrasound findings (Johansen *et al.*, 2024). To date, only limited gwUPD cases have been reported in pregnancy loss, with a frequency ranging from 0.28% to 1.8% (summarized in Supplementary Table S6) (Darcy *et al.*, 2015; Wang *et al.*, 2017, 2020; Maisenbacher *et al.*, 2019; Qu *et al.*, 2019; Kato *et al.*, 2022; Li *et al.*, 2022; Lei *et al.*, 2023; Xue *et al.*, 2023; Chen *et al.*, 2024; Xu *et al.*, 2024; Okonkwo *et al.*, 2025). Our data presented here showed an overall incidence of 1.4% (15/1049) of gwUPD in blastocysts revealed by a concurrent PGT approach, and among these gwUPD embryos with available parent samples, all were of maternal origin (6/6). This finding aligns with a recent study (Picchetta *et al.*, 2025), in which 85.7% (12 of 14) of haploid embryos were predominantly caused by an absence of the paternal genome. This concordance is biologically plausible when considered in the context of early embryogenesis, as gynogenotes (maternal genome-only embryos) are known to be more prevalent than androgenotes (paternal genome-only embryos) in non-induced scenarios.

Furthermore, the parental and segregational origin of triploidy and UPD exerts various clinical significance. Diandric triploidy leads to partial hydatidiform mole, which is a pre-malignant presentation of gestational trophoblastic disease requiring more extensive post-operation care after evacuation surgery (Pan *et al.*, 2021). Moreover, the exclusion of aneuploid embryos alone does not guarantee the embryo’s viability or healthy development. In contrast, the capacity of PGT to identify embryos free of meiotic aneuploidies substantially reduces the likelihood of implantation failure and miscarriage resulting from chromosomal abnormalities (Essers *et al.*, 2023), as meiotic aneuploidies are propagated to all daughter cells of the embryo. Nonetheless, longitudinal studies are mandatory to validate the connection between the segregational origin of aneuploidies and pregnancy outcomes. Additionally, the average maternal and paternal ages of this overall cohort were 36.6 and 39.2 years, respectively. No association was observed between parents’ age and the incidence of gwUPD or triploidy, a finding that might be attributed to the limited number of these two abnormalities.

Nevertheless, there are some limitations inherent to the methodology. Similar to karyomapping and other recently reported reduced representative sequencing methods, PGT-Plus has a continuing need for an additional family member for haplotype construction, although an embryo with a known genotype by prior independent testing is also acceptable. Furthermore, our mosaicism detection focused primarily on copy-number variants in the context of PGT-A, as it is well recognized that embryos with mosaic aneuploidy can still result in healthy live births. As this platform was not designed to detect mosaic forms of gwUPD, this constitutes a limitation of the present study, in light of the reported adverse clinical implications of mosaic gwUPD on pregnancies, including miscarriage or fetal demise (Jawahir-Schonauer *et al.*, 2022).

## Conclusion

In summary, including triploidy and UPD determination in a single concurrent PGT workflow can provide a more comprehensive understanding of the genomic abnormalities in preimplantation embryos to enable improved embryo prioritization. This, in turn, can partially elucidate unexplained implantation failure and miscarriage following the transfer of euploid embryos. This comprehensive approach can expand the diagnostic capabilities of PGT beyond standard methods, distinguish translocation carrier embryos from balanced ones, enhance the overall clinical utility of PGT for infertile couples, and increase treatment benefits for those carrying structural or monogenic disorders, without imposing additional costs or instrument requirements on PGT laboratories.

## Supplementary Material

hoag044_Supplementary_Data

## Data Availability

The datasets are available from the corresponding author on request.
