# Supplementary material for: Underrecognized triploidy and genome-wide uniparental disomy in human blastocysts revealed by a concurrent preimplantation genetic testing approach
Source: Hum Reprod Open. 2026 May 19;2026(3):hoag044. doi: 10.1093/hropen/hoag044 (PMC13262742; doi:10.1093/hropen/hoag044)
Supplement: hoag044_Supplementary_Data [file hoag044_supplementary_data.zip › HRO-25-0369-R2-SupplMaterials and methods.docx]

**Supplementary Materials and methods**

**Sequencing data analysis**

Paired-end reads were aligned to the human reference genome (GRCh37/hg19) using Burrows-Wheeler-Alignment (Li and Durbin, 2009). Uniquely mapped reads were retained for subsequent analysis. Single-nucleotide variants and small insertions/deletions were identified by the Genome Analysis Toolkit (Broad Institute, Cambridge, USA) (McKenna *et al.*, 2010).

***Copy-number profiling based PGT-A analysis***

For trophectoderm biopsies, copy-number variant (CNV) analysis primarily targeted whole-chromosome aneuploidies and segmental aneuploidies exceeding 4 Mb, including mosaic events within a 30%-70% range. This threshold was determined to balance clinical impacts and technical detectability. Firstly, a relatively permissive cutoff was set to maximize the number of embryos classified as transfer-competent, recognizing the potential viability of embryos with lower-level mosaicism. Secondly, this range was selected to enhance diagnostic specificity by reducing technical artifacts, thereby ensuring that reported mosaicism reflects a genuine biological state. The analytical workflow followed a previously reported method (Zhang *et al.*, 2013). Reads amount ≥ 80 M, coefficient of variance < 0.6, logRR_MAPD < 0.15, and logRR_SD < 0.25 were considered passing quality control. Briefly, read counts normalized in 1 Mb sliding windows in test samples were compared against a reference database. Copy-number values were plotted across chromosome coordinates, and genomic segments exceeding 4 Mb with significant deviations from the reference were designated as CNVs, the breakpoints of which were determined by a binary segmentation algorithm (Olshen *et al.*, 2004).

***SNP-based PGT-M/SR linkage analysis***

For linkage analysis in PGT-M and PGT-SR, informative SNPs located within 2 Mb flanking regions (both upstream and downstream) of the target variant or translocation breakpoint were selected to construct parental haplotypes and phase the reference (Zhang *et al.*, 2017). Informative SNPs were defined as positions at which one parent was heterozygous and the other homozygous. Haplotyping required a reference sample from a sibling or other appropriate family member. When such a reference was unavailable, an embryo from the same IVF cycle whose genetic status had been determined by orthogonal targeted testing (e.g., Sanger sequencing) could be used as the reference. Considering the probability of allele dropout, preference was given to an embryo either positive for the monogenic variant in PGT-M or carried unbalanced derivative chromosomes from parental rearrangements in PGT-SR.

***Triploidy and uniparental disomy identification***

Ploidy and heterozygosity status could be identified through the joint analysis of the Z-test, B-allele frequency (BAF), and runs of homozygosity value (ROH). In a normal diploid genome, Z-scores of all chromosomes were within the range of -3 to 3, heterozygous SNP loci exhibited BAF values clustered around 0.5, and ROH values of autosome were around 0. By contrast, triploidy would present with Z-scores ≥ 3 across all chromosomes. In cases of genome-wide UPD (gwUPD), Z-scores would fall ≤ -3 and ROH values would be ≤ -1 throughout the genome, reflecting a dramatic depletion of heterozygosity. Importantly, copy-number loss of a genomic region could also decrease these three metrics, necessitating careful differentiation from UPD.

$Z score=\frac{u(x-x0)}{\sigma2(x0)}$,

where *x* = Percentage of heterozygous SNPs x the means of the difference between the BAF value of each heterozygous SNP and 0.5, *x_0_* = the set of *x* values in the internal reference dataset.

$ROH score=\mathrm{lo}g2\frac{F1}{F0}$,

where F1 = the percentage of heterozygous SNPs within each window in a testing sample, F0 = the percentage of heterozygous SNPs within each window in the internal reference dataset.

***Parental origin and segregational origin analysis for triploidy, UPD, and aneuploidy***

For embryos with numerical or heterozygosity abnormalities, parental and segregational origin were determined based on rulesets previously established (Ariad *et al.*, 2021; McCoy *et al.*, 2015; Rabinowitz *et al.*, 2012). In brief, trisomy and triploidy arising from meiotic errors exhibit two to three distinct parental haplotypes, reflecting contributions from both parental homologs (BPH) from one parent and a third one from the other. Conversely, trisomy and triploidy of mitotic origin show two identical haplotypes from a single parental homolog (SPH) and a third distinct homolog from the other parent. Meanwhile, UPD resulting from monosomy rescue exhibits only a single haplotype.

Furthermore, when centromeric regions originate from different homologs (BPH signature spans the centromere), this indicates a meiotic I error. Alternatively, when centromeric regions correspond to the same homolog (BPH pattern is distal to the centromere), this indicates a meiotic II error where sister chromatids are mal-segregated.

**References**

Ariad D, Yan SM, Victor AR, Barnes FL, Zouves CG, Viotti M, Mccoy RC. Haplotype-aware inference of human chromosome abnormalities. *Proc Natl Acad Sci U S A* 2021;**118**:e2109307118.

Li H, Durbin R. Fast and accurate short read alignment with Burrows-Wheeler transform. *Bioinformatics* 2009;**25**:1754–1760.

McCoy RC, Demko Z, Ryan A, Banjevic M, Hill M, Sigurjonsson S, Rabinowitz M, Fraser HB, Petrov DA. Evidence of Selection against Complex Mitotic-Origin Aneuploidy during Preimplantation Development. *PLoS Genet* 2015;**348**:235–8.

McKenna A, Hanna M, Banks E, Sivachenko A, Cibulskis K, Kernytsky A, Garimella K, Altshuler D, Gabriel S, Daly M, *et al.* The genome analysis toolkit: A MapReduce framework for analyzing next-generation DNA sequencing data. *Genome Res* 2010;**20**:1297–1303.

Olshen AB, Venkatraman ES, Lucito R, Wigler M. Circular binary segmentation for the analysis of array-based DNA copy number data. *Biostatistics* 2004;**5**:557–572.

Rabinowitz M, Ryan A, Gemelos G, Hill M, Baner J, Cinnioglu C, Banjevic M, Potter D, Petrov DA, Demko Z. Origins and rates of aneuploidy in human blastomeres. *Fertil Steril* 2012;**97**:395–401.

Zhang C, Zhang C, Chen S, Yin X, Pan X, Lin G, Tan Y, Tan K, Xu Z, Hu P, *et al.* A Single Cell Level Based Method for Copy Number Variation Analysis by Low Coverage Massively Parallel Sequencing. *PLoS One* 2013;**8**:e54236.

Zhang S, Lei C, Wu J, Zhou J, Sun H, Fu J, Sun Y, Sun X, Lu D, Zhang Y. The establishment and application of preimplantation genetic haplotyping in embryo diagnosis for reciprocal and Robertsonian translocation carriers. *BMC Med Genomics* 2017;**10**:1–9.
