# Supplementary material for: Underrecognized triploidy and genome-wide uniparental disomy in human blastocysts revealed by a concurrent preimplantation genetic testing approach
Source: Hum Reprod Open. 2026 May 19;2026(3):hoag044. doi: 10.1093/hropen/hoag044 (PMC13262742; doi:10.1093/hropen/hoag044)
Supplement: hoag044_Supplementary_Data [file hoag044_supplementary_data.zip › HRO-25-0369-R2-Table1_EO.docx]

**Table 1. Significant roles of triploidy and uniparental disomy in various sample cohorts**

|  | **PGT category** | **Maternal/**  **Paternal age (means)** | **Sample size** | | **Triploidy** | | **Uniparental disomy** | | |
| --- | --- | --- | --- | --- | --- | --- | --- | --- | --- |
|  |  |  | **No. of embryos** | **No. of cycles** | **No. of embryos** | **No. of cycles** | | **No. of**  **embryos** | **No. of cycles** |
| **Phase II:**  **Retrospective clinical validation** | **PGT-A** | 37.7/40.0 | 130 | 66 | 2 | 2 | | 2 (gwUPD) | 2 |
|  | **PGT-M+A** | 34.4/37.0 | 65 | 8 | 0 | 0 | | 2 (gwUPD, mat) | 2 |
|  | **PGT-SR+A** | 33.8/37.9 | 67 | 9 | 0 | 0 | | 1 (gwUPD, mat) | 1 |
| **Subtotal-1** | | 37.0/39.5 | 262 | 83 | 2 (0.8%) | 2 (2.4%) | | 5 (1.9%) | 5 (6.0%) |
| **Phase III:**  **Prospective diagnostic implementation** | **PGT-A** | 37.8/40.0 | 320 | 84 | 2 | 2 | | 1 (UPD18) | 1 |
|  | **PGT-M+A** | 33.7/37.5 | 146 | 28 | 1 | 1 | | 2 (gwUPD, mat) | 2 |
|  | **PGT-SR+A** | 33.7/36.7 | 63 | 11 | 0 | 0 | | 1 (gwUPD, mat) | 1 |
| **Subtotal-2** | | 36.5/39.1 | 529 | 123 | 3 (0.6%) | 3 (2.4%) | | 4 (0.8%) | 4 (3.3%) |
| **A special cohort: euploid embryos transferred with adverse outcomes ^a^** | | 37.0/39.2 | 258 | 202 | 3 (1.2%) | 3 (1.5%) | | 6 (2.3%, gwUPD) | 6 (3.0%) |
| **Total** | | 36.8/39.2 | 1049 | 408 | 8 (0.8%) | 8 (2.0%) | | 15 (1.4%) | 15 (3.7%) |

^a^includes not being pregnant, biochemical pregnancy and miscarriage.

gwUPD, genome-wide uniparental disomy; mat, maternal origin; PGT-A/M/SR, preimplantation genetic testing for aneuploidy / monogenic disorder / structural rearrangement.
