# Supplementary material for: Underrecognized triploidy and genome-wide uniparental disomy in human blastocysts revealed by a concurrent preimplantation genetic testing approach
Source: Hum Reprod Open. 2026 May 19;2026(3):hoag044. doi: 10.1093/hropen/hoag044 (PMC13262742; doi:10.1093/hropen/hoag044)
Supplement: hoag044_Supplementary_Data [file hoag044_supplementary_data.zip › Supp.figures_EO.docx]

**
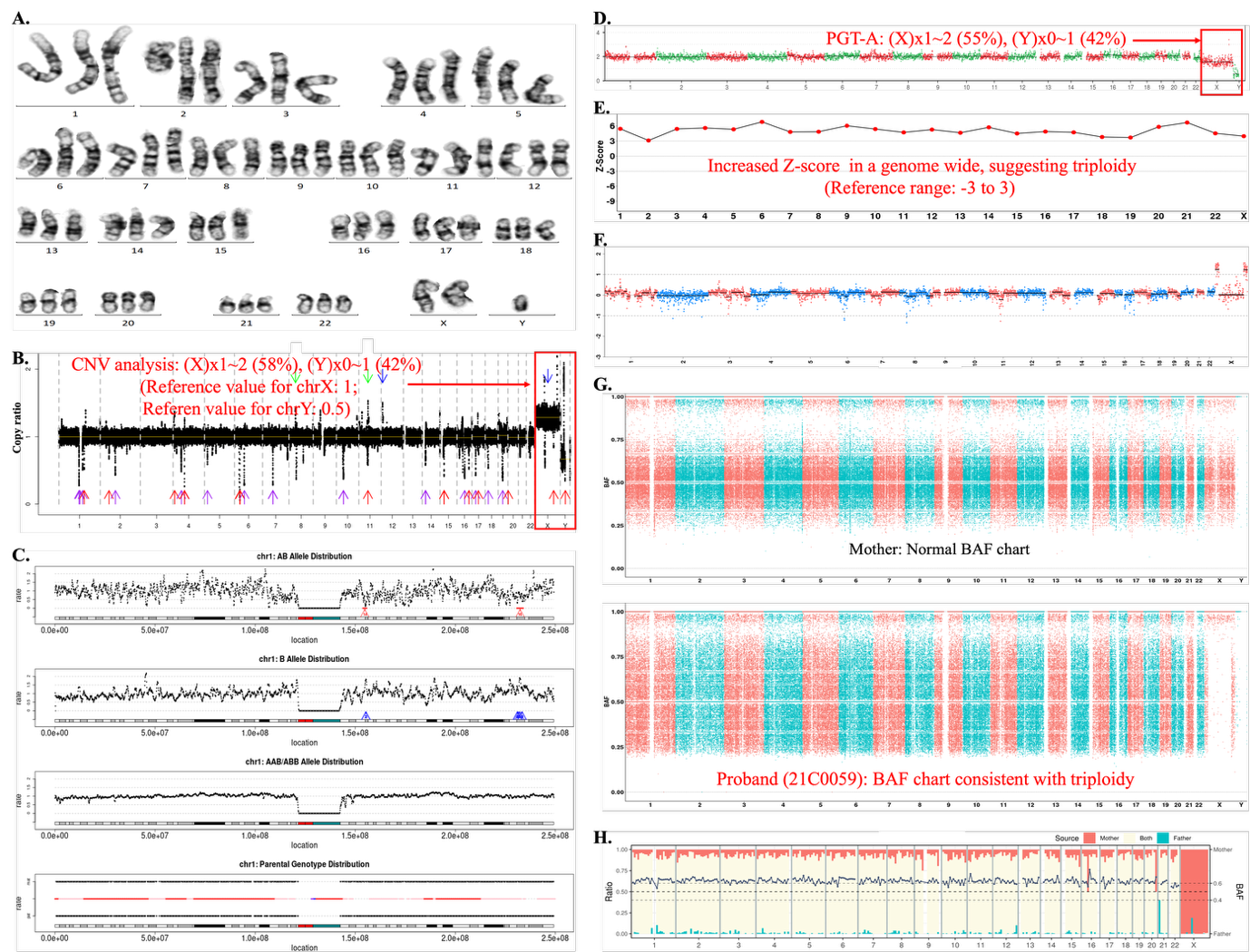
**

**Supplementary Figure S1: Maternally originated triploidy of miscarried villi in Phase I, aligned with prior results.** (A) Karyogram: All chromosomes had three copies at 550 banding level, diagnostic of triploidy. (B and C) Mate-pair genome sequencing results of gDNA. (B) Copy-number profile: Chr X showed an approximate 50% increase and chrY a 50% decrease in copy-number relative to a diploid male reference, while no copy-number abnormalities detected in autosomes. This profile is consistent with 69,XXY karyotype. (C) Heterozygosity analysis (shown for chr1 as an example): The first to third panels indicate AOH. The fourth panel shows excess of maternal genotypes (red line, maternal/paternal genotype ratio >5). Collectively, these findings suggest that the extra chromosome sets is of maternal origin. (D-H) PGT-Plus results of this MDA-amplified gDNA sample (21C0059). (D) Copy-number profile: The proband showed sex chromosome dosage imbalance consistent with 69,XXY. (E-G) Triploidy indicated by ploidy analysis based on heterozygous SNPs. This is characterized by elevated (> 3) Z-Scores across all chromosomes (E) and typical BAF graph (G). (H) Parental origin analysis: The curved line skewed shift towards maternal contribution (BAF value 0.5-0.75), suggesting maternal origin of the extra chromosome set (Reference value: 0.5).

MDA, multiple displacement amplification; gDNA, genomic DNA; AOH, absence of homozygosity; ROH, runs of homozygosity; BAF, b-allele frequency; Chr, chromosome.

**
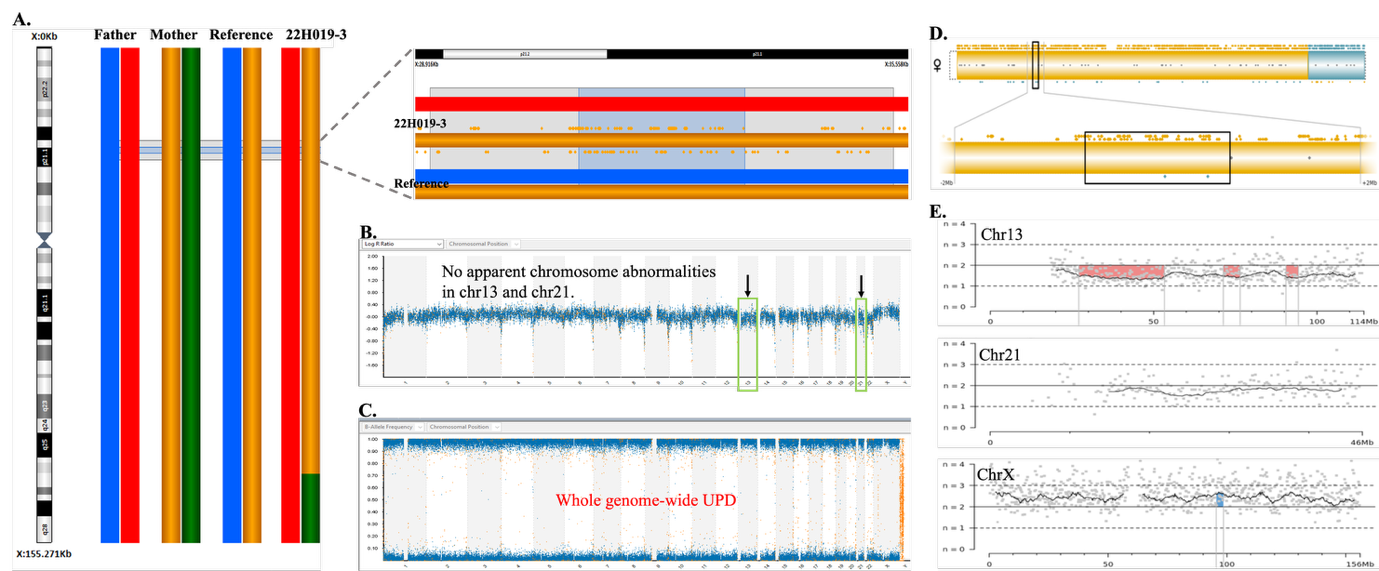
**

**Supplementary Figure S2:** **PGT-M and PGT-A results of one representative genome-wide uniparental disomy case by prior PGT platforms.** (A-C) Results of embryo 22H019-3 by conventional Karyomapping. (A) Haploblocks of informative SNPs for the *DMD* gene on chromosome X: Maternal chromosomes are visualized in green and yellow, and paternal chromosomes in blue and red. The target region is highlighted in blue and grey shades. Key SNPs (strong phase evidence) and non-key SNPs (weaker evidence) are plotted above and below the haploblock bar, respectively. All SNPs supported that embryo 22H019-3 inherited the mutant maternal allele in the *DMD* gene. (B) Log R ratio: No overt chromosomal abnormality was detected by Karyomapping, consistent with its limited sensitivity for mosaicism detection. (C) BAF: Despite the neutral copy-number profile, however, this embryo was identified as having gwUPD. (D-E) Results of embryo 22H019-3 by prior concurrent platform OnePGT (Agilent). (D) Haplotype analysis of the target region: The black box denotes the target region. Shared haploblocks with the sibling (in phase) are depicted in yellow, whereas non-shared ones are shown in turquoise. Informative SNPs supporting the assigned haploblocks are positioned above each chromosome, while discordant SNPs are shown below. No paternal chrX. Therefore, embryo 22H019-3 inherited the mutant maternal phase. (E) PGT-A analysis by OnePGT: Mosaic monosomy 13 and a 3 Mb mosaic segmental gain on chrX were detected, findings largely consistent with PGT-Plus results.

**
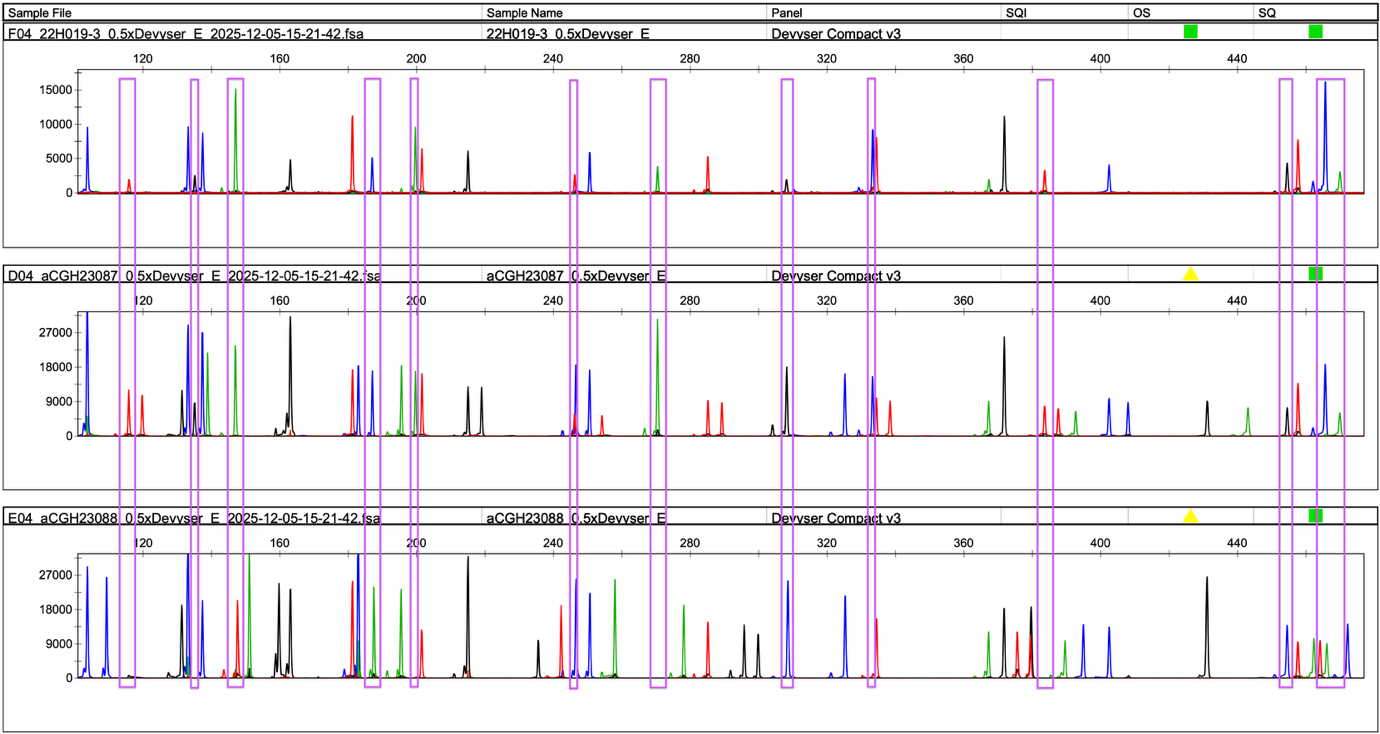
**

**Supplementary Figure S3: Orthogonal validation of maternally originated genome-wide uniparental disomy by qfPCR: A representative case demonstration.** STR profiles for a representative trio: embryo 22H019‑3 (top), mother aCGH23087 (middle), and father aCGH23088 (bottom) were presented. Of the 26‑marker panel, 14 informative STR loci (highlighted in purple) unambiguously supported maternal origin of the gwUPD, as the embryo’s alleles at these loci were identical to those of the mother and discordant with the father. Genotype analysis across these 14 informative polymorphic loci on five chromosomes excluded paternal contribution. The peak size of all STR markers, both informative and non-informative, are provided in Supplementary Table S4. Other results of this case are provided in Fig. 2.

gwUPD, Genome-wide uniparental disomy; qfPCR, quantitative fluorescent PCR; STR, Short tandem repeats.


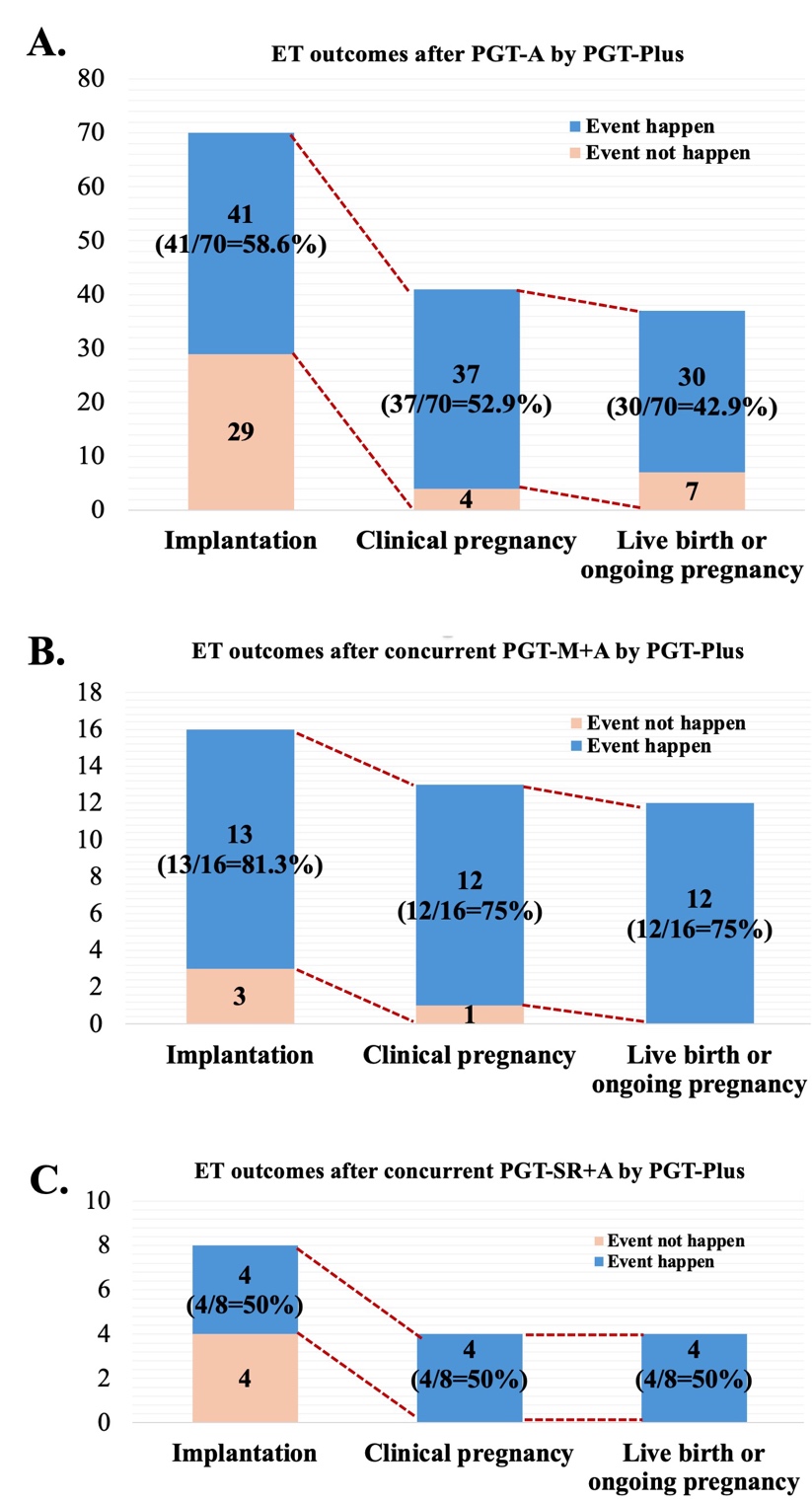


**Supplementary Figure S4: Embryo transfer outcomes based on PGT-Plus findings in Phase III prospective cohort.** (A) Outcomes following PGT-A by PGT-Plus: Of 70 transferred embryos, 41 implanted successfully (blue, left), while 29 were implantation failures (orange, left). Of the implanted embryos, 37 progressed to clinical pregnancy with fetal heartbeat confirmed by ultrasound (blue, middle), while 4 resulted in biochemical pregnancy (orange, middle). Of the clinical pregnancies, 30 led to live birth or ongoing pregnancy (blue, right), while 7 ended in miscarriage (orange, right). (B) Outcomes following concurrent PGT-M+PGT-A by PGT-Plus: Of 16 transferred embryos, 13 implanted (blue, left) and 3 failed to implant (orange, left). Among implanted embryos, 12 developed into a clinical pregnancy (blue, middle), and one was biochemical pregnancy (orange, middle). All clinical pregnancies progressed to live birth or ongoing pregnancy (blue, right). (C) Outcomes following concurrent PGT-SR+PGT-A by PGT-Plus: Of 8 transferred embryos, 4 implanted and 4 failed to implant. All implanted embryos developed into clinical pregnancy and subsequently progressed to live birth or ongoing pregnancy. No miscarriages occurred.
