# Supplementary material for: Underrecognized triploidy and genome-wide uniparental disomy in human blastocysts revealed by a concurrent preimplantation genetic testing approach
Source: Hum Reprod Open. 2026 May 19;2026(3):hoag044. doi: 10.1093/hropen/hoag044 (PMC13262742; doi:10.1093/hropen/hoag044)
Supplement: hoag044_Supplementary_Data [file hoag044_supplementary_data.zip › Supplementary tables_clean_20260324.docx]

**Supplementary Table S1.** **Prenatal and/or postnatal cells with known triploidy or AOH/UPD used for technical validation in Phase I**

| **Sample ID** | **Genetic complement from prior testing platform(s)** | **Results from PGT-Plus** | | | | |
| --- | --- | --- | --- | --- | --- | --- |
|  |  | **CNV** | **Ploidy** | | **AOH/UPD** | |
| 20C2082 | ChromoSeq:  seq (X)x2,(Y)x1,  (1-22)x3 (pat) | seq (X)x3[0.51],(Y)x0[0.49] | | Triploidy  (pat) | | Not detected |
| 21C0059 | ChromoSeq:  seq (X)x2,(Y)x1,  (1-22)x3 (mat) | seq (X)x3[0.55],(Y)x0[0.42] | | Triploidy (mat) | | Not detected |
| 21C1197 | ChromoSeq:  seq (X)x2,(Y)x1,  (1-22)x3 (pat) | seq (X)x3[0.36],(Y)x0[0.28] | | Triploidy  (pat) | | Not detected |
| 20C1503 | Karyotyping: 69,XXY | seq (X)x3[0.5],(Y)x0[0.54] | | Triploidy | | Not detected |
| 21C3001 | Karyotyping: 69,XXY | seq[GRCh37] (X)x3[0.28],(Y)x0[0.52],  10q11.21q22.1(42670001_72850000)x3[0.57],  10q22.1q23.33(72220001_96200000)x3,  10q23.33q26.3(95700001_135530000)x3[0.55],  (19)x3[0.5],  (22)x3[0.47] | | Triploidy | | Not detected |
| 21C2506 | Karyotyping: 69,XXX | seq (X,1-22)x2 | | Triploidy | | Not detected |
| 21C1219 | Karyotyping: 69,XXX | seq (X,1-22)x2 | | Triploidy | | Not detected |
| 21C2504 | Karyotyping: 70,XXY,+10 | seq (X)x3[0.67],(Y)x0[0.57],(10)x3 | | Triploidy | | Not detected |
| 20C2251 | Karyotyping: 70,XXY,+6 | seq (6)x3[0.68],(X)x3[0.43],(Y)x0[0.65],(6)x3[0.68] | | Triploidy | | Not detected |
| 24C1758 | ChromoSeq:  seq (X,1-22)x2hmz (pat) | seq (X,1-22)x2 | | Diploidy | | Genome-wide paternal isoUPD |
| 20C0963 | FetalChip: Extended homozygosity of 408 Mb on 11 chromosomes (1,2,3,6,9,11,12,  13,14,15,20) | seq (1-22)x2 | | Diploidy | | seq[GRCh37]  Xq27.2q28(142000001_148000000)x2hmz,  1p21.1p12(102500001_120500000)x2hmz,  2q21.2q24.3(133500001_166500000)x2hmz,  2q31.1q36.3(173000001_226500000)x2hmz,  3p12.3q13.2(76000001_113500000)x2hmz,  3p14.1p12.3(69000001_75000000)x2hmz,  3p22.3p22.1(34000001_43500000)x2hmz,  5p15.33p15.32(10001_6000000)x2hmz,  6p25.2p22.3(4000001_21500000)x2hmz,  6q15q23.3(91500001_136000000)x2hmz,  7p22.3p22.1(10001_6500000)x2hmz,  9q22.33q33.1(100000001_120000000)x2hmz,  11q14.1q23.1(78000001_111000000)x2hmz,  12p13.33p13.31(10001_10000000)x2hmz,  12q24.21q24.33(116500001_130500000)x2hmz,  13q12.3q13.3(31500001_37000000)x2hmz,  13q22.2q31.3(75500001_93500000)x2hmz,  13q33.1q34(102000001_115159878)x2hmz,  14q24.3q31.3(78500001_86000000)x2hmz,  15q11.2q13.3(22500001_32500000)x2hmz,  15q14q15.2(36000001_43500000)x2hmz,  18p11.31p11.21(5500001_11500000)x2hmz,  20p12.3p11.23(6000001_21000000)x2hmz,  20q13.13q13.31(47500001_56500000)x2hmz  Xq27.2q28(142000001_148000000)x2hmz |
| 20C1951 | FetalChip:  269 Mb AOH (chr1-3, 5-7,12-14 & 17) | seq (1-22)x2 | | Diploidy | | seq[GRCh37] 1q23.3q31.1(164500001_186000000)x2hmz,  1q32.1q42.13(207000001_227500000)x2hmz,  2p12p11.2(75000001_87500000)x2hmz,  2p25.2p24.3(5000001_15500000)x2hmz,  2q32.3q33.3(192500001_208500000)x2hmz,  3q26.31q29(173000001_193500000)x2hmz,  5q12.1q13.2(62500001_69500000)x2hmz,  5q35.2q35.3(173000001_180905260)x2hmz,  6q21q25.1(110500001_152500000)x2hmz,  7p15.2p14.3(27500001_34000000)x2hmz,  7q31.1q33(113500001_134500000)x2hmz,  8q24.13q24.22(125500001_133500000)x2hmz,  10q22.1q23.1(72500001_84000000)x2hmz,  10q23.2q24.1(88500001_98000000)x2hmz,  10q25.3q26.11(115000001_120500000)x2hmz,  12q24.31q24.33(121000001_133841895)x2hmz,  13q33.2q34(105500001_115159878)x2hmz,  14q22.1q23.1(51000001_61500000)x2xhmz,  15q12q14(26500001_34000000)x2hmz,  16p13.3p13.12(10001_14500000)x2hmz,  17q21.2q25.2(38500001_75000000)x2hmz,  22q13.31q13.33(45500001_51294566)x2hmz |
| 20C2031 | FetalChip:  78 Mb AOH  (chr 7,12,16 &17) | seq (1-22)x2 | | Diploidy | | seq[GRCh37] 7q22.1q31.1(103500001_111500000)x2hmz,  12q21.33q24.11(91000001_110000000)x2hmz,  12q24.13q24.23(112500001_119000000)x2hmz,  16q21q23.1(63500001_76000000)x2hmz,  17p12p11.2(14000001_20500000)x2hmz,  17q11.1q12(25500001_36000000)x2hmz |
| 21C2967 | ChromoSeq: seq[GRCh37] (X,Y)x1,(1-22)x2,  9p24.3p24.1(200000_5300000)x2hmz | seq (1-22)x2 | | Diploidy | | seq[GRCh37] 9p24.3p24.1(10001_5000000)x2hmz |

Note:

ChromoSeq and FetalChip are lab-developed tests which have been applied in clinical diagnosis, which are mate-pair genome sequencing and chromosomal microarray, respectively.

Abbreviations:

aCGH: array comparative genome hybridization; AOH: absence of heterozygosity; hmz: homozygosity; Mat: maternal origin; Pat: paternal origin.

**Supplementary Table S2.** **Family and disease information of concurrent PGT-M and PGT-A cycles from both Phase II and Phase III**

|  | **Gene (OMIM ID)** | **Cytogenetic location** | **Phenotype** | **Inheritance mode** | **Number of embryos** |
| --- | --- | --- | --- | --- | --- |
| Retrospective | *HBA1* & *HBA2*  (*141800 &*141850) | 16p13.3 | α-thalassemia | AR | 30 (4 cycles of 4 couples) |
|  | *MNX1* (*142994) | 7q36.3 | Currarino syndrome | AD | 10 (1 cycle) |
|  | *ABCD1* (*300371) | Xq28 | X-Linked Adrenoleukodystrophy | XLR | 10 (1 cycle) |
|  | *DMD* (*300377) | Xp21.2p21.1 | Duchenne muscular dystrophy | XLR | 4 (1 cycle) |
|  | *GLB1* (*611458) | 3p22.3 | GM1-gangliosidosis | AR | 11 (1 cycle) |
| Prospective | *HBA1* & *HBA2*  (*141800 & *141850) | 16p13.3 | α-thalassemia | AR | 12 (3 cycles of 2 couples) |
|  | *HBB* (*141900) | 11p15.4 | β-thalassemia | AR | 11 (2 cycles of 2 couples) |
|  | *FBN1* (*134797) | 15q21.1 | Marfan syndrome | AD | 7 (1 cycle) |
|  | *RBM20* (*613171) | 10q25.2 | Dilated cardiomyopathy | AD | 3 (1 cycle) |
|  | *F8* (*300841) | Xq28 | Hemophilia A | XLR | 18 (2 cycles of 2 couples) |
|  | *KLHL40* (*615340) | 3p22.1 | Nemaline myopathy 8 | AR | 6 (2 cycles of 1 couple) |
|  | - | 16p11.2 | 16p11.2 deletion syndrome | AD | 3 (1 cycle) |
|  | *FSHD1* (*158900) | 4q35 | Facioscapulohumeral muscular dystrophy 1 | AD | 12 (3 cycles of 2 couples) |
|  | *NF1* (*613113) | 17q11.2 | Neurofibromatosis, type 1 | AD | 4 (1 cycle) |
|  | *SERPINA1* (*107400) | 14q32.13 | Alpha-1 antitrypsin deficiency | AR | 20 (2 cycles of 1 couple) |
|  | *PKD1* (*601313) | 16p13.3 | Polycystic kidney disease 1 | AD | 3 (1 cycle) |
|  | *PKD2* (*173910) | 4q22.1 | Polycystic kidney disease 2 | AD | 10 (3 cycles of 2 couples) |
|  | - | 22q11.21 | 22q11.2 deletion syndrome | AD | 17 (2 cycles of 1 couple) |
|  | *EXT1* (*608177) | 8q24.11 | Hereditary multiple osteochondromas | AD | 4 (1 cycle) |
|  | *GLB1* (*611458) | 3p22.3 | GM1-gangliosidosis | AR | 8 (1 cycle) |
|  | *SMN1* (*600354) | 5q13.2 | Spinal muscular atrophy | AR | 8 (2 cycles of 2 couples) |
| Total | | | | | 211 (36 cycles of 30 couples) |

Abbreviations:

AD: autosomal dominant; AR: autosomal recessive; OMIM: Online Mendelian Inheritance in Man; XLR: X-linked recessive.

**Supplementary Table S3.** **Family and disease information of concurrent PGT-SR and PGT-A cycles from both Phase II and Phase III**

| **Family** | **Translocation and carrier family member** | **Reference** | **Number of embryos**  **(number of cycles)** |
| --- | --- | --- | --- |
| Retrospective | Maternal: 46,XX,t(6;10)(q27;q22.3) | No real reference family member, an unbalanced translocation embryo according to prior PGT-A platform was designated as the reference. | 7 (1 cycle) |
|  | Maternal: 46,XX,t(12;15)(q23;q24) |  | 11 (1 cycle) |
|  | Maternal: 46,XX,t(14;20)(q32.2;q11.2) |  | 12 (1 cycle) |
|  | Maternal: 46,XX,t(3;7)(q28;q35) |  | 10 (1 cycle) |
|  | Paternal: 46,XY,t(15;20)(q21.2;q12) |  | 6 (1 cycle) |
|  | Paternal: 46,XY,t(3:19)(p21.3;q13.3) |  | 13 (3 cycles of 1 couple) |
|  | Paternal: 46,XY,t(1;4)(q32.3;q31.3) |  | 8 (1 cycle) |
| Prospective | Maternal: 46,XX,t(4;14)(q27;q32.33) | Maternal grandmother | 4 (2 cycles of 1 couple) |
|  | Maternal: 45,XX,der(13;14)(q10;q10) | Maternal grandfather | 4 (1 cycle) |
|  | Maternal: 46,XX,t(10,11)(q23.2;q21) | Maternal grandfather | 12 (2 cycles of 1 couple) |
|  | Paternal: 46,XY,t(6;9)(p21.3;q13) | Daughter | 11 (1 cycle) |
|  | Maternal: 46,XX,t(5;7)(p15.3;p21) | Previous chorionic villus sampling sample | 3 (1 cycle) |
|  | Paternal: 46,XY,ish t(1;8)(p36.32;q24.3)  (RP11-1143\|12+;RP11-846C3+) | Amniotic fluid sample | 16 (2 cycles of 1 couple) |
|  | Paternal: 46,XY,t(1;3)(p13;p11),t(5;9)(p15.2;p21) | No real reference, an unbalanced embryo (due to parental translocation) was designated as the reference. | 3 (1 cycle) |
|  | Maternal: 45,XX,der(14;21)(q10;q10) |  | 10 (1 cycle) |
| Total | | | 130  (20 cycles of 15 couples) |

**Supplementary Table S4. Genotypes by orthogonal qfPCR validation in a representative genome-wide uniparental disomic trio**

| **Marker** | **Fluorescent dye** | **Chromosome loci** | **Embryo:**  **Peak size (bp)** | **Mother:**  **Peak size (bp)** | **Father:**  **Peak size (bp)** | **Inheritance** |
| --- | --- | --- | --- | --- | --- | --- |
| 13A_D13S742 | Green | 13q12.12 | **270** | **270** | 258/278 | Maternally inherited |
| 13B_D13S634 | Blue | 13q21.32q21.33 | 402 | 402/408 | 400/402 | Non-informative |
| 13C_D13S628 | Black | 13q31.1 | **455** | 431/**455** | 431 | Maternally inherited |
| 13D_D13S305 | Green | 13q13.3 | **470** | 443/**470** | 462/466 | Maternally inherited |
| 13K_D13S1492 | Red | 13q21.1 | **116** | **116**/120 | 148 | Maternally inherited |
| 18B_D18S978 | Black | 18q12.3 | 215 | 215/219 | 215 | Non-informative |
| 18C_D18S535 | Blue | 18q12.3 | **333** | 325/**333** | 309/325 | Maternally inherited |
| 18D_D18S386 | Green | 18q22.1 | 367 | 367/393 | 367/390 | Non-informative |
| 18J_D18S976 | Red | 18p11.31 | 458 | 458 | 458/464 | Non-informative |
| 18M_GATA178F11 | Black | 18p11.32 | 372 | 372 | 372/380 | Non-informative |
| 21A_D21S1435 | Blue | 21q21.3 | **187** | 183/**187** | 183 | Maternally inherited |
| 21B_D21S11 | Blue | 21q21.1 | 251 | 247/251 | 247/251 | Non-informative |
| 21C_D21S1411 | Black | 21q.23.3 | **308** | **308** | 296/300 | Maternally inherited |
| 21D_D21S1444 | Blue | 21q22.13 | **466** | **466** | 454/472 | Maternally inherited |
| 21H_D21S1442 | Red | 21q21.3 | **384** | **384**/388 | 376/379 | Maternally inherited |
| 21I_D21S1437 | Black | 21q21.1 | **135** | 131/**135** | 131 | Maternally inherited |
| AMELXY | Blue | Xp22.2/Yp11.2 | 104 | 104 | 104/109 | Non-informative |
| SRY | Black | Yp11.31 | No detected alleles | No detected alleles | 236 | Non-informative |
| T1 | Red | 7q34/Xq13 | 181/**202** | 181/**202** | 181/201 | Maternally inherited |
| T3 | Blue | 3p24.2/Xq21.1 | 133/137 | 133/137 | 133/137 | Non-informative |
| X1_DXS1187 | Green | Xq26.2 | **147** | 139/**147** | 151 | Maternally inherited |
| X3_XHPRT | Red | Xq26.2q26.3 | 285 | 285/289 | 285 | Non-informative |
| X9_DXS2390 | Red | Xq27.1q27.2 | 334 | 334/338 | 334 | Non-informative |
| XY2_DXYS267 | Green | Xq21.3/Yp11.31 | **200** | 195/**200** | 187/195 | Maternally inherited |
| XY3_DXYS218 | Red | Xp22.33/Yp11.32 | **246** | **246**/254 | 242 | Maternally inherited |
| ZFYX | Black | Yp11.31/Xp22.11 | 163 | 163 | 160/163 | Non-informative |

Note: Inheritance of informative genotypes is highlighted in bold font.

**Supplementary Table S5. Cost and turn-around time analysis of the PGT-Plus approach**

|  | **PGT-Plus** | **OnePGT**  **(Agilent)** | **Karyomapping (Vitrolife)** | **VeriSeq (Illumina)** |
| --- | --- | --- | --- | --- |
| **WGA method** | MDA or PicoPLEX or SurePLEX | MDA | MDA | SurePLEX or PicoPLEX |
| **Sequencer/Scanner** | MGI2000 or DA500 | Illumina NextSeq500 | iScan system | Illumina Miseq |
| **Samples per run** | Maximum 32 or 36 | PGT-M: 24  PGT-A/PGT-SR: 96 | 12 | 24 |
| **Cost** | **+** | **+++** | **++++** | **++** |
| **Turn-around time** | **+** | **++++** | **++** | **++** |
| **Detection scope, advantages and disadvantages** | 1) Simultaneous detection and analysis of PGT-A/M/SR;  2) Triploidy, AOH, UPD (both UPiD and UPhD).  Specifically:  PGT-A:  1) Aneuploidy;  2) ≥4 Mb del/dup;  3) Mosaicism ≥ 30% (aneuploidies & ≥10 Mb CNVs);_  PGT-M:  Relative haplotype phasing;  PGT-SR:  1) Distinguish between normal and balanced carrier;  2) Cryptic SR less than 4 Mb. | 1) Aneuploidy;  2) ≥ 5 Mb del/dup;  3) Determination of the origin of aneuploidy;  4) UPD. | 1) PGT-M;  2) PGT-A: Suboptimal resolution for segmental aneuploidy and mosaicism;  3) UPiD;  4) Cannot distinguish monosomy and UPD. | Only PGT-A:  1) Aneuploidy;  2) ≥ 10 Mb del/dup;  3) Mosaicism ≥ 30% (aneuploidy & ≥ 10 Mb CNVs)._ |

Abbreviations:

AOH: absence of heterozygosity; CNV: copy-number variant; Del/dup: deletion/duplication; MDA: multiple displacement amplification; UPhD: uniparental heterodisomy; UPiD: uniparental isodisomy.

**Supplementary Table S6. Contribution of genome-wide uniparental disomy in miscarriages previously reported**

| **Journal** | **Platform** | **Sample size** | **No. of gwUPD** | **Percentage** | **Reference** |
| --- | --- | --- | --- | --- | --- |
| *J Clin Med.* | CNV-seq + NGS-Based self-designed STR panel Test | 500 | 9 | 1.80% | (Lei *et al.*, 2023) |
| *Clin. Genet.* | SNP array | 535 | 8 | 1.50% | (Wang *et al.*, 2017) |
| *Reprod Med Biol.* | NGS +QF‐PCR | 300 | 2 | 0.70% | (Kato *et al.*, 2022) |
| *Ultrasound Obstet Gynecol.* | CMA | 3101 | 19 | 0.61% | (Wang *et al.*, 2020) |
| *J. Matern. Fetal Neonatal Med.* | SNP array | 484 | 3 | 0.60% | (Qu *et al.*, 2019) |
| *Appl Clin Genet.* | CMA | 1220 | 4 | 0.33% | (Xu *et al.*, 2024) |
| *Clin. Chim. Acta.* | CNV-seq + QF-PCR | 4211 | 13 | 0.31% | (Chen *et al.*, 2024) |
| *Fertil. Steril.* | SNP array | 22451 | 72 | 0.30% | (Maisenbacher *et al.*, 2019) |
| *Sci Rep.* | SNP array | 711 | 2 | 0.28% | (Xue *et al.*, 2023) |
| *Mol Cytogenet.* | SNP array | Case report | 1 | - | (Okonkwo *et al.*, 2025) |
| *Am J Med Genet A.* | SNP array + STR analysis | Case report | 1  (mosaic gwUPD) | - | (Darcy *et al.*, 2015) |
| Reprod. Sci. | CNV-seq + STR analysis | Case report | 1 | - | (Li *et al.*, 2022) |

Abbreviations:

CMA: chromosomal microarray; CNV: copy number variants; gwUPD: genome-wide uniparental disomy; NGS: next-generation sequencing; QF-PCR: quantitative fluorescence polymerase chain reaction; SNP: single nucleotide polymorphism; STR: short-tandem repeats.

**References:**

Chen Y, Han X, Hua R, Li N, Zhang L, Hu W, Wang Y, Qian Z, Li S. Copy number variation sequencing for the products of conception: What is the optimal testing strategy. *Clinica Chimica Acta* 2024;**557**.

Darcy D, Atwal PS, Angell C, Gadi I, Wallerstein R. Mosaic paternal genome-wide uniparental isodisomy with down syndrome. *Am J Med Genet A* 2015;**167**:2463–2469.

Kato T, Miyai S, Suzuki H, Murase Y, Ota S, Yamauchi H, Ammae M, Nakano T, Nakaoka Y, Inoue T, *et al.* Usefulness of combined NGS and QF-PCR analysis for product of conception karyotyping. *Reprod Med Biol* 2022;**21**.

Lei C, Liao K, Zhao Y, Long Z, Zhu S, Wu J, Xiao M, Zhou J, Zhang S, Li L, *et al.* A Novel System for the Detection of Spontaneous Abortion-Causing Aneuploidy and Its Erroneous Chromosome Origins through the Combination of Low-Pass Copy Number Variation Sequencing and NGS-Based STR Tests. *J Clin Med* 2023;**12**.

Li M wei, Li F, Cheng J, Wang F, Zhou P. Recurrent Androgenetic Complete Hydatidiform Moles with p57KIP2-Positive in a Chinese Family. *Reproductive Sciences* 2022;**29**:1749–1755.

Maisenbacher MK, Merrion K, Kutteh WH. Single-nucleotide polymorphism microarray detects molar pregnancies in 3% of miscarriages. *Fertil Steril* 2019;**112**:700–706. Available at: https://doi.org/10.1016/j.fertnstert.2019.06.015.

Okonkwo OO, Ortega V, Kane S, Aldrete G, Ramirez P, Valente PT, Velagaleti GVN. Whole genome uniparental isodisomy detected using single nucleotide polymorphism (SNP) microarray in molar pregnancy: a case report. *Molecular Cytogenetics* 2025;**18**.

Qu S, Wang L, Cai A, Cui S, Bai N, Liu N, Kong X. Exploring the cause of early miscarriage with SNP-array analysis and karyotyping. *The Journal of Maternal-Fetal & Neonatal Medicine* 2019;**32**:1–10. Available at: https://doi.org/10.1080/14767058.2017.1367379.

Wang Y, Cheng Q, Meng L, Luo C, Hu H, Zhang J, Cheng J, Xu T, Jiang T, Liang D, *et al.* Clinical application of SNP array analysis in first-trimester pregnancy loss: a prospective study. *Clin Genet* 2017;**91**:849–858.

Wang Y, Li Y, Chen Y, Zhou R, Sang Z, Meng L, Tan J, Qiao F, Bao Q, Luo D, *et al.* Systematic analysis of copy‐number variations associated with early pregnancy loss. *Ultrasound in Obstetrics & Gynecology* 2020;**55**:96–104.

Xu Z, Liu N, Gao L, Yu D. Application of Chromosomal Microarray Analysis in Genetic Reasons of Miscarriage Tissues. *Application of Clinical Genetics* 2024;**17**:85–93.

Xue H, Guo Q, Yu A, Lin M, Chen X, Xu L. Genetic analysis of chorionic villus tissues in early missed abortions. *Sci Rep* 2023;**13**.
